# Association Between Early Sexual Debut and New HIV Infections Among Adolescents and Young Adults in 11 African Countries

**DOI:** 10.1007/s10461-024-04343-w

**Published:** 2024-06-15

**Authors:** Jiawei He, Abraham Flaxman, Jeffrey W. Imai-Eaton, Aleksandr Aravkin, Peng Zheng, Reed Sorensen, Shachi Mittal, Hmwe H. Kyu

**Affiliations:** 1 Institute for Health Metrics and Evaluation, University of Washington, Seattle, USA; 2 Department of Health Metrics Sciences, School of Medicine, University of Washington, Seattle, USA; 3 Center for Communicable Disease Dynamics, Department of Epidemiology, Harvard T.H. Chan School of Public Health, Boston, USA; 4 MRC Centre for Global Infectious Disease Analysis, School of Public Health, Imperial College London, London, UK; 5 Department of Chemical Engineering, University of Washington, Seattle, USA

**Keywords:** Early sexual debut, New HIV infection, Adolescents and young adults, PHIA surveys, African countries

## Abstract

We investigated the association between early sexual debut and HIV infection among adolescents and young adults. Analyzing data from nationally representative Population-Based HIV Impact Assessment (PHIA) surveys in 11 African countries, the research employed a multivariate logistic regression model to assess the relationship between the early sexual debut and new HIV infections in the age group of 10–24 years. The results revealed a significant and robust association, indicating that young individuals who experienced early sexual debut were approximately 2.65 times more likely to contract HIV than those who did not, even after accounting for other variables. These findings align with prior research suggesting that early initiation of sexual activity may increase vulnerability to HIV infection due to factors such as biological susceptibility and risky behaviors like low condom use and multiple sexual partners. The implications of these findings for HIV prevention strategies are substantial, suggesting that interventions aimed at delaying sexual debut could be an effective component in reducing HIV risk for this population. Targeted sex education programs that address the risks of early sexual debut may play a pivotal role in these prevention efforts. By employing a comprehensive approach, there is a possibility to advance efforts towards ending AIDS by 2030.

**Supplementary Information:**

The online version contains supplementary material available at 10.1007/s10461-024-04343-w.

## Introduction

HIV infection continues to pose a significant public health challenge globally, with adolescents and young women being particularly vulnerable [18]. This demographic group, aged between 10 and 24 years, is often at a heightened risk due to a combination of biological, behavioral, and structural factors [41]. One behavioral factor which has been the subject of considerable research and debate is the age of sexual debut. Early sexual debut is typically defined as engaging in sexual intercourse for the first time at a young age. Early sexual debut, defined as sexual initiation before the age of 18 years [8, 19], has been linked to poor sexual health outcomes and lower overall well-being in some studies [45]. Demographic and Health Surveys (DHS) conducted in Latin America, the Caribbean, and sub-Saharan Africa have shown the percentage of early sexual debut to range from 12 to 30% between 2013 and 2016 [13].

Growing evidence has shown that early sexual debut is associated with risk factors of HIV infections, including multiple lifetime partners, inconsistent condom use, and a history of sexual violence [33, 49]. Early sexual debut may increase a young person’s risk for HIV infection and sexually transmitted infections (STIs), such as through forced sex and having older partners [32, 49]. However, few studies have built the research based on a conceptual framework or mechanism through which this increased risk may occur. One study did not consider the relationship between covariates, exposure, and outcome from a causal perspective, and might have controlled for the mediators that ought not to be controlled for, such as the duration of sexual activity, having multiple partners, and engaging in other risky behaviors (e.g. lower rates of using condoms) [49, 58]. While these covariates can be associated with higher risks of acquiring HIV infection, they are the mediators between early sexual debut and HIV infection because early sexual debut can influence these risk factors but not the other way around. They are on the potential causal pathways through which early sexual debut can cause HIV infections.

One systematic review [56] identified some potential pathways by which early sexual debut could increase the risk of HIV infections in girls, including biological HIV susceptibility due to the immature reproductive tract and risk behaviors correlated with early sexual debut such as multiple sexual partners and low condom use. Importantly, these potential pathways do not function independently. They are embedded within a wider socio-cultural and economic context characterized by gender disparities and societal norms. Built on this conceptual framework, this study aims to quantify the association between early sexual debut and the risk of recent HIV infection for adolescents and young adults, including exploring sex differences in these associations, across countries in Africa using the Population-Based HIV Impact Assessment (PHIA) surveys.

## Methods

### Data Collection

The PHIA [29] surveys are cross-sectional household-based surveys implemented by the International Center for AIDS Care and Treatment Programs (ICAP) at Columbia University. The surveys were conducted in 14 countries in Sub-Saharan Africa (SSA), as displayed in Appendix Table A.1, from 2015 to 2019 and were designed to collect standardized data across countries.

Eleven countries are included in this study.^1^ The majority of the questions asked during interviews in different countries are identical. The description of the countries with available data used in the analysis is displayed in Table 1. In this study, a total of 99,672 records were included, comprising 55,249 females and 44,423 males. Among these records, there were 2687 prevalent cases and 104 incident infections identified.

**Table 1:** Descriptive statistics

Country	Overall	Female	Male	10–17	18–24	Prevalent cases	New infections
Cameroon	11,123	6004 (53.98%)	5119 (46.02%)	5133 (46.1%)	5990 (53.9%)	127 (1.14%)	11 (0.099%)
Côte d’Ivoire	5917	3169 (53. 56%)	2748 (46.44%)	1783 (30.1%)	4134 (69.9%)	38 (0.64%)	3 (0.051%)
Eswatini	4740	2496 (52.66%)	2244 (47.34%)	2414 (50.9%)	2326 (49.1%)	382 (8.06%)	13 (0.274%)
Ethiopia	7522	4780 (63.55%)	2742 (36.45%)	1937 (25.8%)	5585 (74.2%)	62 (0.82%)	1 (0.013%)
Lesotho	5505	3061 (55.6%)	2444 (44.4%)	2762 (50.2%)	2743 (49.8%)	373 (6.78%)	13 (0.236%)
Malawi	6233	3563 (57.16%)	2670 (42.84%)	1732 (27.8%)	4501 (72.2%)	215 (3.45%)	7 (0.112%)
Namibia	7396	3889 (52.58%)	3507 (47.42%)	3498 (47.3%)	3898 (52.7%)	278 (3.76%)	11 (0.149%)
Rwanda	11,358	6070 (53.44%)	5288 (46.56%)	3640 (32%)	7718 (68%)	103 (0.91%)	2 (0.018%)
Tanzania	10,299	5799 (56.31%)	4500 (43.69%)	3336 (32.4%)	6963 (67.6%)	171 (1.66%)	4 (0.039%)
Uganda	12,948	7216 (55.73%)	5732 (44.27%)	5387 (41.6%)	7561 (58.4%)	242 (1.87%)	14 (0.108%)
Zambia	7301	4151 (56.86%)	3150 (43.14%)	2148 (29.4%)	5153 (70.6%)	290 (3.97%)	15 (0.205%)
Zimbabwe	9330	5051 (54.14%)	4279 (45.86%)	4809 (51.5%)	4521 (48.5%)	406 (4.35%)	10 (0.107%)
Total	99,672	55,249 (55.43%)	44,423 (44.57%)	38,579 (38.71%)	61,093 (61.29%)	2687 (2.7%)	104 (0.104%)

The sample sizes are presented for the columns ‘Overall’, ‘Female’, ‘Male’, ‘10–17’, and ‘18–24’. The ‘Prevalent cases’ and ‘New infections’ columns show the number of cases. The male and female numbers are aggregated across all age groups, while the numbers for each age group represent both sexes. In the analysis, a few countries (Ethiopia, Côte d’Ivoire, Tanzania) with no available data on early sexual activity in the age group 10–14 are excluded from modeling. Rwanda has a different weighting scheme, and thus not included in modeling

### Framework

Stockl [56] has proposed potential causal pathways that influence women’s early sexual debut and its effect on the risk of HIV infection. Prior studies have found that early sexual debut is associated with increased sexual risks including multiple sexual partners and using a condom irregularly [28, 54]. Gender can be associated with different social norms and expectations regarding sexual behavior [46]. Females initiate sexual activity younger than males [46]. Education, wealth, and periurban residence (vs rural) have been associated with age of sexual debut [40]. Adolescents with more education may evaluate the health and financial implications of smaller families differently, leading to increased likelihood of avoiding unwanted pregnancy and reducing their risk of HIV/AIDS through delayed sexual activity [22]. Urban versus rural residence is associated with differences in access to healthcare, cultural values, and living conditions, all of which are associated with sexual debut age [22]. Specifically, living in a more rural area is often associated with earlier age of first sex [31]. These sociodemographic factors are also associated with the risks of HIV infections.

**Fig. 1:**
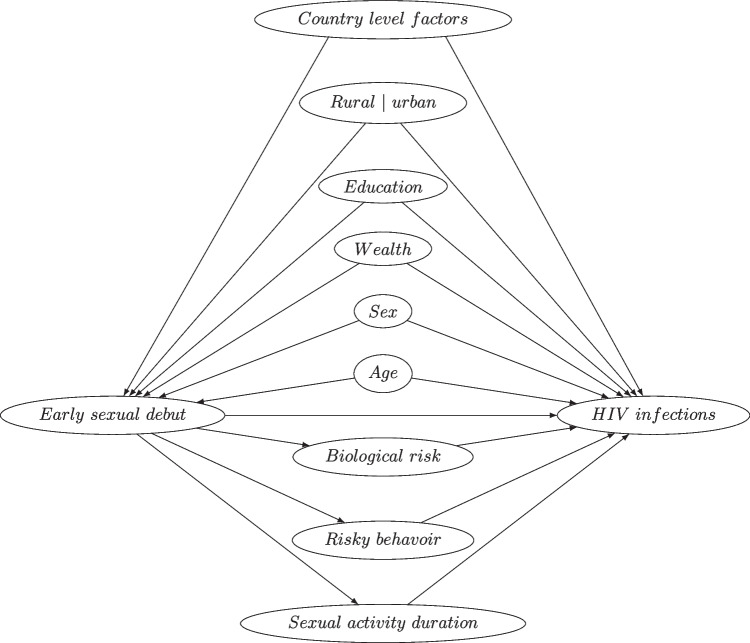
Conceptual framework of factors influencing early sexual debut and its relationship with increased risk of HIV infection

The relationships between these factors are displayed in Fig. 1. In our study, these confounders will be controlled for in the model. These covariates are discussed in more detail in the Appendix. Some country level factors like cultural norms (e.g. attitudes and beliefs towards sex) and public health programs (e.g. parental education programs) are also associated with both early sexual debut and HIV infections [41]. The country variable will be included in the model as fixed effects to capture the uncontrolled country level factors.

### Modeling

The primary outcome of interest in this study is the recent acquisition of HIV infection. The covariates included in this study encompass early sexual debut, age, sex, education level, place of residence, household wealth, and country of residence. For this research, we impute the missing data by employing the multiple imputation by chained equations (MICE). MICE is a statistical method widely used in health science to handle missing data in a dataset, and has been proven to outperform other imputation techniques in some simulation experiments [11, 53]. We implement the imputation of missing data using the MICE package [57] in the R statistical software.

The statistical analysis for this study involves the implementation of multivariate logistic regression models, adjusted to account for the survey sampling design. These models are employed to examine the associations between early sexual debut and HIV infection among both adolescents and young adults. The models control for potential confounding variables and incorporate a fixed effect to account for variability at the country level.

The measures of association are expressed in terms of odds ratios (OR) along with their corresponding 95% confidence intervals (CI). These measures provide an estimate of the strength and precision of the relationship between early sexual debut and HIV infections. The computational platform for these analyses is the statistical programming language R, with the use of the survey package [42], which is specifically designed for analyzing data from complex surveys.

#### Sensitivity Analysis

Sensitivity analysis allows for the exploration of uncertainties and the assessment of the overall robustness of the research [12]. In addition, sensitivity analysis can play a crucial role in validating models throughout the development processes [37]. The sensitivity analysis in this research will specifically explore nine hyperparameters to assess the study’s robustness: the number of sets of data imputations, imputation algorithms, whether to include data of young adolescents (10–14 years), different cutoff ages for early sexual debut, gender-specific models, modeling the interaction between gender and early sexual debut, model country-level HIV prevalence as a covariate, handle observations that are censored, and the imputation methods. The sets of sensitivity analyses are shown in Table 2.

**Table 2:** Sets of sensitivity analysis

	Hyperparameters	Benchmark model	Alternative scenarios
1	Number of imputation sets	5 sets	10, 20, 50 sets
2	Imputation algorithms	Predictive mean matching	Random sample, classification and regression trees, Bayesian linear regression
3	Inclusion of young adolescents (10–14 years)	Included	Excluded
4	Cutoff age for early sexual debut	18 years	16, 17 years
5	Gender-specific models	Males and females combined	Males and females separately
6	Interaction between gender and early sexual debut	Not included	Included
7	Country-level HIV prevalence as covariate	Not included	Included (drop country variable)
8	Handling censoring	Included	Excluded
9	Imputation methods	MICE	Amelia

#### Population Attributable Fraction

**Fig. 2:**
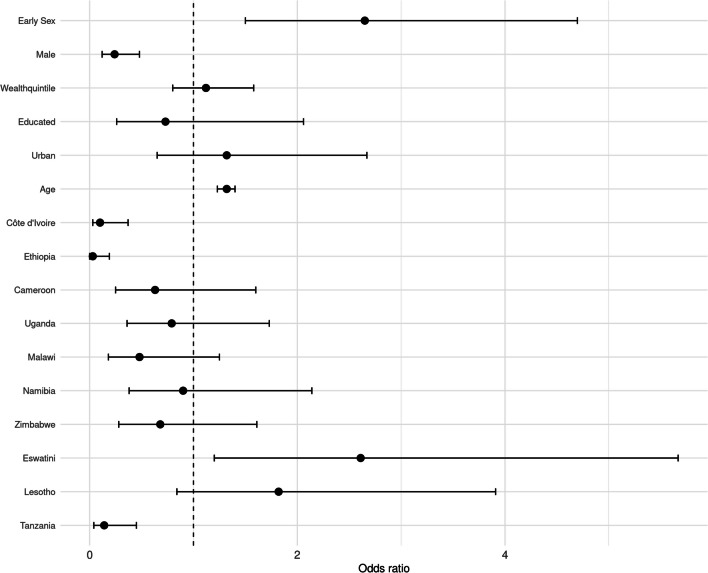
Odds ratio (OR) and 95% confidence intervals (CI) from the baseline model. *Note* The reference country for the country variable is Zambia

In the study, we calculate the PAF to quantify the proportion of HIV infections that could be prevented if early sexual debut were eliminated in the age group of 10–24 years for both males and females across different countries. Specifically, we estimate the PAF using the following formula,

$$\begin{aligned} PAF = \frac{Prev\cdot (OR-1)}{1 + Prev\cdot (OR-1)} \end{aligned}$$

where *Prev* is the prevalence of early sexual debut in each country. *OR* is the odds ratio of early sexual debut from the model as shown in Fig. 2.

To account for the uncertainties in the model, we have incorporated the variability of both the prevalence of early sexual debut and the odds ratio associated with early sexual debut into our estimates of the PAF through the Monte Carlo simulation approach. The details can be found in the Online Appendix.

## Results

### Descriptive Analyses

The estimates of HIV prevalence taking into account the sampling weights among individuals aged 10 to 24 are displayed in Appendix Figure B.1, including both the aggregate and sex-specific estimates. These estimates reveal considerable variations across different countries and genders. Côte d’Ivoire records the lowest overall prevalence rate at 0.63%, whereas Eswatini reports the highest prevalence at 7.41%. A consistent pattern emerges across all countries, where female populations demonstrate a higher HIV prevalence compared to their male counterparts. These observations align with the findings from prior research [17, 26].

Online Appendix Fig. B.2 shows the annual estimated incidence rate^2^ of HIV for each country, including both overall and sex-specific estimates. The observed patterns are similar to those found in the prevalence estimates. There is substantial heterogeneity in incidence across both countries and genders. Lesotho and Eswatini report the highest incidence rate. Furthermore, in all countries—except Ethiopia and Côte d’Ivoire—female populations exhibit higher incidence compared to males.^3^

In Online Appendix Fig. B.3, we present the country-specific prevalence rates of early sexual debut among individuals aged 10 to 24, stratified by gender. It shows that males consistently have a higher proportion of early sexual debut compared to females across all countries except Ethiopia. The data reveal considerable inter-country variability; for instance, Lesotho has the highest observed prevalence among males, exceeding 50%, whereas Zimbabwe, Ethiopia, and Eswatini report prevalence below 15%.

### Multivariate Analyses

The results of the logistic regression model, incorporating the survey sampling design and controlling for various covariates, are presented in Fig. 2 and Online Appendix Table C.1. The estimated odds ratios (ORs) indicate the variation in the likelihood of acquiring an HIV infection corresponding to a one-unit increase in each explanatory variable, holding all other covariates constant.

The findings from the baseline model demonstrate that early sexual debut is associated with significantly higher odds of HIV infection among adolescents and young adults. Specifically, those engaging in early sexual activity are approximately 2.65 times more likely to have recent HIV infections compared to their counterparts who do not debut sexual activity before age 18, given that all other variables remain unchanged (95% CI [1.5, 4.7]).

Gender was significantly associated with recent HIV infection, with males demonstrating around 76% lower odds of recent HIV infection compared to females (OR 0.24, 95% CI [0.12, 0.48]). Age also plays a substantial role, with each additional year correlating with an approximately 32% increase in the odds of recent HIV infection (OR 1.32, 95% CI [1.23, 1.40]). However, the associations between recent HIV infection and wealth quintile, educational status, and urban residence are not statistically significant at the 95% confidence level.

The estimated ORs for the country-specific variables are interpreted relative to Zambia, the reference category. For example, residing in Ethiopia is associated with a 96% lower odds of recent HIV infection compared to Zambia. Conversely, residents of Eswatini had significantly higher risk of recent HIV infection, with 2.6 times the odds of acquiring HIV compared to Zambia (OR 2.60, 95% CI [1.20, 5.66]).

### Summary of Sensitivity Analyses

We conducted an extensive range of sensitivity analyses to evaluate the robustness of our model. These analyses are detailed in Online Appendix Figs. D.1 to D.9 and Appendix Tables D.1 to D.9.

Firstly, we found that varying the number of data imputations did not significantly alter the model’s conclusions, as shown in Online Appendix Fig. D.1. Similarly, the use of different imputation algorithms yielded consistent results, confirming our model’s robustness (Online Appendix Fig. D.2).

We also examined the impact of including young adolescents in the study and found that the model’s conclusions remained stable regardless of their inclusion (Online Appendix Fig. D.3). This stability extended to varying the cutoff ages for sexual debut between 16 and 18 years (Online Appendix Fig. D.4).

In terms of gender-specific models, we observed that the effect of early sexual debut was significant for females but not for males, which could be attributed to reduced statistical power (Online Appendix Fig. D.5). However, including an interaction term for gender and sexual debut did not significantly change the model’s conclusions (Online Appendix Fig. D.6).

We also considered the inclusion of country-level HIV prevalence as a covariate and found that, while it did not change the overall conclusions, it was associated with a 10% higher odds ratio for HIV infection (Online Appendix Fig. D.7).

Lastly, our model proved to be robust to the handling of censored data (Online Appendix Fig. D.8) and was consistent across different imputation methods, such as Amelia and MICE (Online Appendix Fig. D.9).

Overall, our model’s conclusions are robust to various sensitivity analyses.

### Population Attributable Fractions

**Fig. 3:**
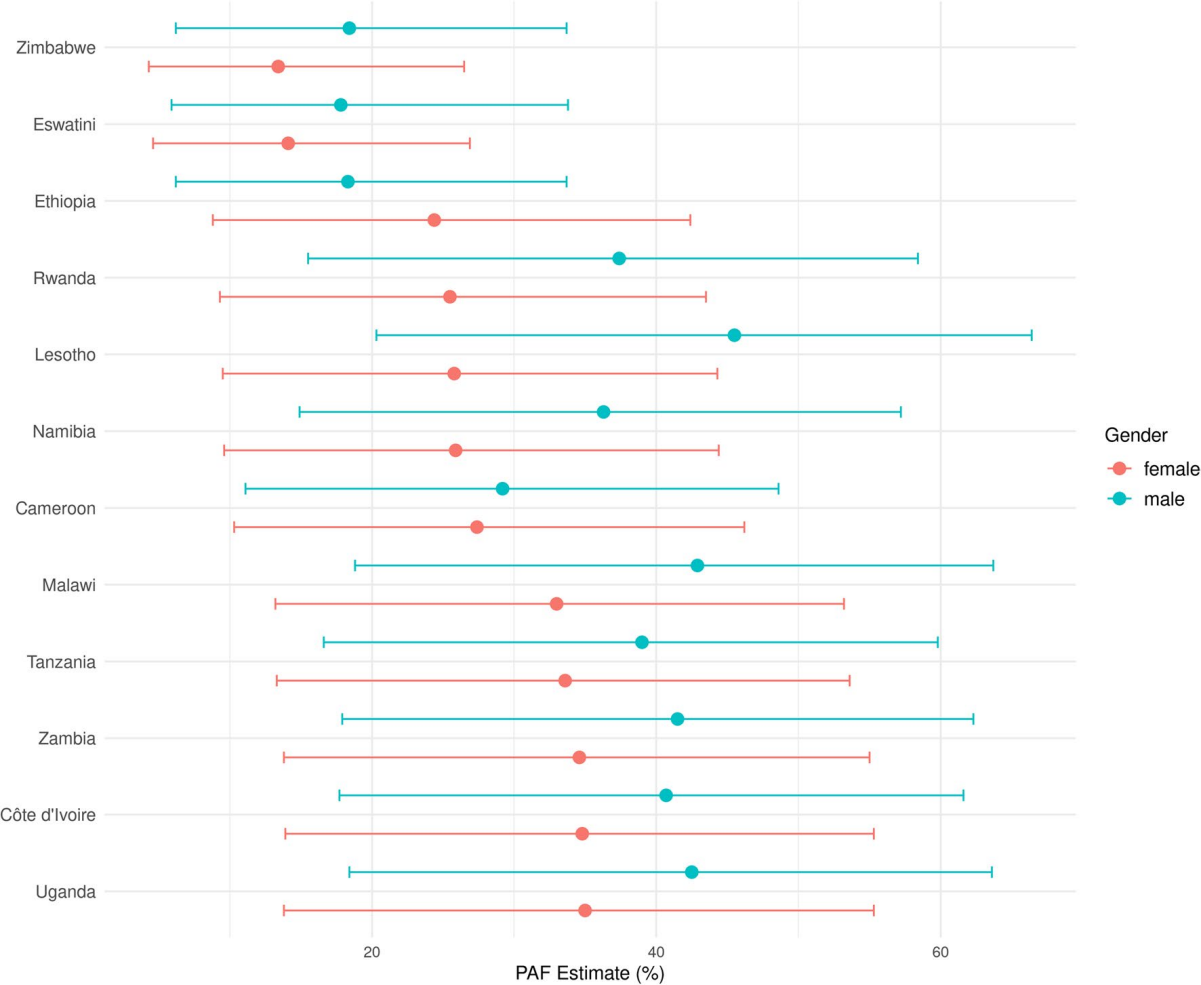
PAF for males and females aged 10–24 years in each country

The PAF of HIV infections attributable to early sexual debut for males and females aged 10–24 years in each country is shown in Fig. 3. A striking observation from the results is the consistently higher PAF values for males compared to those for females across all countries under study (Note that the PAFs for males have larger uncertainty intervals.) This is likely due to the higher prevalence of early sexual debut among males in all countries. This suggests that early sexual debut is a more substantial contributing factor to HIV infections for young males than for young females. Notably, Lesotho has the highest PAF for males recorded at 45.6% (95% CI [20.5% to 66.4%]) and Uganda has the highest PAF for females recorded at 34.9% (95% CI: [13.9% to 55.2%]). These elevated PAFs are reflective of the high prevalences of early sexual debut among young males and females in these two countries.

## Discussion

The results of this study provide substantial support for the hypothesis that early sexual debut is associated with a higher risk for HIV infection among adolescents and young adults. The multivariate logistic regression model found a strong association between the age of sexual debut and new HIV infections in the age group of 10–24 years. Specifically, young individuals who have an early sexual debut are approximately 2.65 times more likely to contract HIV than those who do not, assuming all other variables are held constant. These findings align with some previous studies suggesting that early initiations of sexual activity may be more vulnerable to HIV infection due to factors such as biological HIV susceptibility due to the immature reproductive tract and risky behaviors such as low condom use and a higher number of sexual partners [24, 33].

The robustness of these findings is further reinforced through sensitivity analyses. These analyses considered the number of data imputations, imputation methods, whether to include young adolescents, the cutoff age for early sexual debut, gender-specific models, interaction between gender and early sexual debut, country-level HIV prevalence as covariate, and handling censoring. The consistency of the results across these different parameters underscores the validity of the study’s results and provides additional confidence in the association between early sexual debut and increased HIV risk. The gender-specific models demonstrate that the impact of sexual debut is more substantial among females than among males.

These findings have important implications for HIV prevention strategies targeting adolescents and young adults. They suggest that interventions aimed at delaying sexual debut could be an effective component of a comprehensive approach to reducing HIV risk for this population. Comprehensive sex education programs, for instance, have shown promise in not only improving knowledge about sexual health, but also in delaying sexual debut [16, 38]. These programs often provide a nuanced understanding of contraception, consent, and the risks associated with early sexual activity. Cash transfer programs have been proven effective in delaying sexual initiation by increasing school enrollment and educational attainment [23].

However, it is also important to note that early sexual debut is just one of many factors that can influence HIV risk. Other factors, such as condom use, number of sexual partners, and substance use, also play a significant role [39, 43]. Therefore, while addressing early sexual debut is crucial, it should not be the sole focus of HIV prevention efforts. A comprehensive approach that addresses multiple risk factors is more likely to be the most effective [14, 25]. Furthermore, the findings of this study highlight the importance of considering the broader social and structural factors that can influence the age of sexual debut. Factors such as wealth, education, and gender inequality can all play a role in determining when young people begin sexual activity. Therefore, efforts to delay sexual debut and reduce HIV risk must also address these broader social determinants of health.

This study also has its limitations. First, relying on self-reported data for sexual debut and other variables may introduce reporting bias. Participants may not accurately recall or may intentionally misreport the age of their sexual debut due to social desirability bias or recall bias. This could potentially lead to misclassification and bias the results. Second, missing data, although imputed, could introduce bias if the data were not missing at random. While we used multiple imputation to handle missing data, the results might be biased if the missingness is related to both the predictors and the outcome. The sensitivity analysis of the data imputation, however, could somewhat mitigate this limitation. Third, the education level variable is dichotomized into “any” or “no” education based on the assumption that the majority under 15 years old would not have completed middle school. This dichotomization might have oversimplified the complexity of educational attainment and its potential effects on the outcomes studied. Finally, the study’s findings may not be generalizable to all adolescents and young adults, as the study population is limited to those aged 10–24 in the countries included in the PHIA survey. The HIV risk associated with early sexual debut may differ in other countries or among different age groups.

Despite these limitations, our study offers several contributions to the existing literature. Unlike previous studies that have primarily focused on populations aged 15 and above, our research uniquely incorporates data from younger adolescents aged 10–14, which are uniquely available through new PHIA surveys. This inclusion provides a more comprehensive understanding of sexual behaviors and associated risks from an earlier age, filling a critical gap in the research. Moreover, while earlier studies with national surveys have been confined to examining prevalent HIV cases, our study is the first of its kind to investigate the association between early sexual debut and new HIV infections rather than relying solely on existing cases. Additionally, our study leverages nationally representative surveys from multiple African countries, enabling a cross-cultural examination of early sexual debut and HIV risk. This approach enhances the generalizability of our findings and allows for a more comprehensive understanding of the relationship between early sexual debut and HIV risk across diverse cultural and socioeconomic contexts. In addition, the robustness of our findings is bolstered by the inclusion of 9 sets of sensitivity analyses. These analyses serve to validate the primary results by examining the impact of various hyper-parameters and assumptions, such as the cutoff age for early sexual debut, gender-specific models, and the inclusion of country-level HIV prevalence as a covariate. This approach enhances the study’s credibility and makes a strong case for its implications in shaping public health policies aimed at reducing HIV risk among adolescents and young adults in diverse African settings.

To further the impact of this research, we have calculated the Population Attributable Fraction of HIV infections associated with early sexual debut for each country using the model results. The PAF estimates serve as a useful metric for public health policy, providing quantifiable evidence that can guide resource allocation and intervention strategies. By understanding the proportion of HIV infections that could potentially be prevented by delaying sexual debut, policymakers and healthcare providers can better target their prevention and intervention efforts. This is particularly crucial in Africa, where HIV prevalence is among the highest globally, and early sexual debut is a common phenomenon.

### Supplementary Information

Below is the link to the electronic supplementary material.

Supplementary file 1 (pdf 3659 KB)

## Footnotes

1 There are no data available for Kenya and Haiti. The young adolescents of Ethiopia and Cote d’Ivoire are not included due to lack of indicator of early sexual debut. The young adolescents of Tanzania and Rwanda are not included due to large proportion of missingness for the indicator of early sex (more than 93%). In addition, Rwanda has a different way of estimating the sampling weights.

2 The annual incidence is calculated using the methods proposed by Kassanjee [34] after taking into account assumed mean duration of recent infection (MDRI).

3 Given that Ethiopia and Côte d’Ivoire also have the lowest incidence rate, these comparisons might be susceptible to bias.
