# Supplementary material for: Association Between Early Sexual Debut and New HIV Infections Among Adolescents and Young Adults in 11 African Countries
Source: AIDS Behav. 2024 Jun 15;28(7):2444–53. doi: 10.1007/s10461-024-04343-w (PMC11199287; doi:10.1007/s10461-024-04343-w)
Supplement: Supplementary file 1 — (pdf 3659 KB) [file 10461_2024_4343_MOESM1_ESM.pdf]

Supplementary Appendix to ”Association between early sexual debut and recent HIV infections among adolescents and young adults in 11 African countries”

TABLE OF CONTENTS

Page

Appendix A: Data description 2

Appendix B: Estimated prevalence and incidence rate 4

Appendix C: Statistical analysis 10
- C.1 Covariates 10
- C.2 Data imputation 11
- C.3 Modeling 13

Appendix D: Sensitivity analysis 16

Appendix E: Population attributable fractions 32

Appendix A

DATA DESCRIPTION

PHIA uses a stratified multistage survey sampling design[1], with strata defined by sub-national geographic divisions. Within each stratum, census enumeration areas (EAs) are randomly selected with probability proportional to population size in the first stage, followed by a random sample of households within selected EAs in the second stage. The consenting households are offered a household interview. In each selected household, individuals are given a structured questionnaire, and individuals who complete the individual interviews are administered biomarker testing. The sampling weights of observations are calculated from sample selection probabilities and adjusted for non-response and post-stratification based on age and sex according to the national population projections from the survey year. The Jackknife replicate weights are provided for variance estimates.

Table A.1: Data collection dates of the countries in PHIA Surveys.

Country	Data Collection Dates
Zimbabwe	Oct 2015 – Aug 2016
Malawi	Nov 2015 – Aug 2016
Zambia	Mar 2016 – Aug 2016
Uganda	Aug 2016 – Mar 2017
Eswatini	Aug 2016 – Mar 2017
Lesotho	Nov 2016 – May 2017
Namibia	Jun 2017 – Nov 2017
Cameroon	Jun 2017 – Jan 2018
Cote d'Ivoire	Aug 2017 – Mar 2018
Ethiopia	Oct 2017 – Apr 2018
Tanzania	Nov 2016 – Jun 2017
Kenya	May 2018 – Mar 2019
Rwanda	Oct 2018 – Mar 2019
Haiti	Jul 2019 - Nov 2020

Appendix B

ESTIMATED PREVALENCE AND INCIDENCE RATE

Figure B.1: The overall prevalence of HIV among people aged 10 - 24 years old by sex and country.

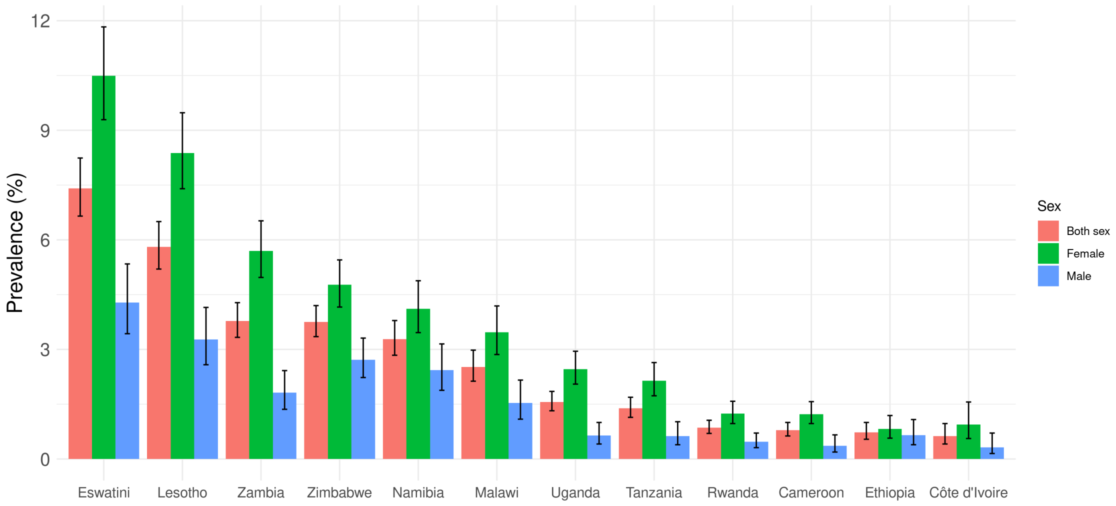

Figure B.2: The incidence rate of HIV among people aged 15 - 24 years old by sex and country.

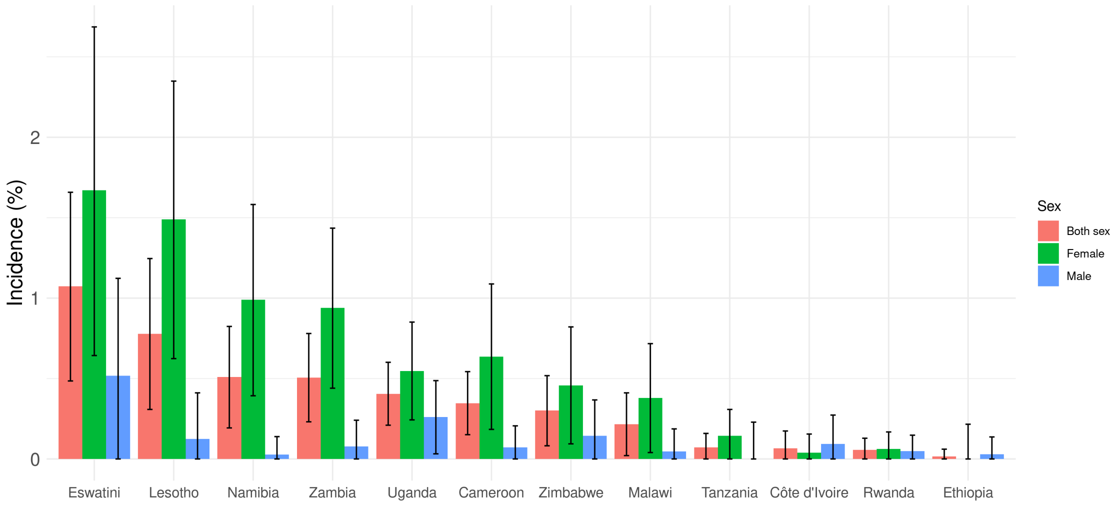

Note: The incidence estimate of 10 - 14 years old in most countries is 0; thus they are not included here.

Figure B.3: The prevalence of early sexual debut among people aged 10 - 24 years old by sex and country.

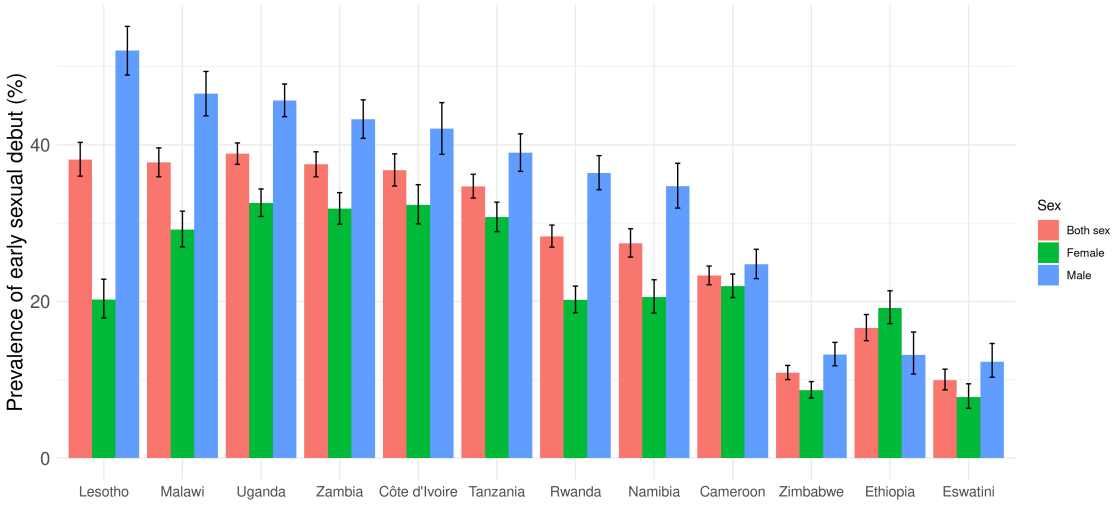

Figure B.4: The prevalence of HIV among people aged 10 - 24 years old by age group and country.

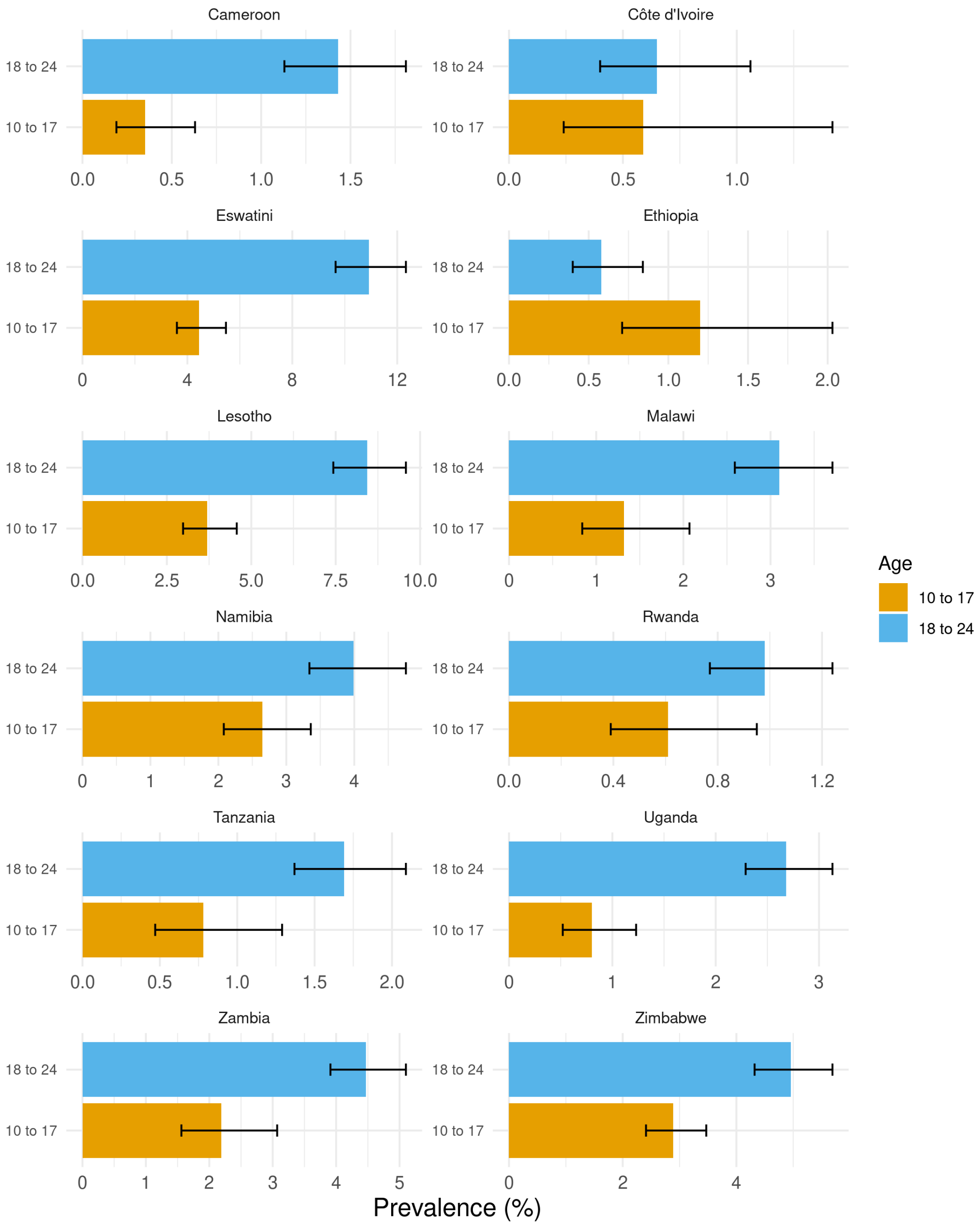

Note: In the analysis, a few countries (Ethiopia, Côte d'Ivoire, Tanzania) with no available data on early sexual activity in the age group 10 - 14 are excluded from modeling.

Table B.1: Prevalence of HIV among people above 15·

Country	Total	Male	Female	10 to 17	18 to 24
Cameroon	0·79 (0·63,1)	0·36 (0·19,0·66)	1·23 (0·97,1·57)	0·35 (0·19,0·63)	1·43 (1·13,1·81)
Côte d'Ivoire	0·63 (0·41,0·97)	0·32 (0·15,0·71)	0·94 (0·56,1·56)	0·59 (0·24,1·42)	0·65 (0·4,1·06)
Eswatini	7·41 (6·65,8·24)	4·28 (3·43,5·34)	10·49 (9·29,11·83)	4·44 (3·6,5·47)	10·92 (9·65,12·33)
Ethiopia	0·73 (0·54,1)	0·65 (0·39,1·08)	0·82 (0·57,1·19)	1·2 (0·71,2·03)	0·58 (0·4,0·84)
Lesotho	5·81 (5·2,6·5)	3·27 (2·58,4·15)	8·38 (7·4,9·48)	3·69 (2·98,4·57)	8·44 (7·43,9·58)
Malawi	2·52 (2·13,2·98)	1·53 (1·09,2·16)	3·47 (2·86,4·19)	1·32 (0·84,2·07)	3·1 (2·59,3·71)
Namibia	3·28 (2·84,3·79)	2·43 (1·88,3·15)	4·11 (3·46,4·88)	2·65 (2·08,3·36)	3·99 (3·34,4·76)
Rwanda	0·86 (0·7,1·06)	0·47 (0·31,0·71)	1·24 (0·97,1·58)	0·61 (0·39,0·95)	0·98 (0·77,1·24)
Tanzania	1·39 (1·14,1·69)	0·63 (0·39,1·02)	2·14 (1·73,2·64)	0·78 (0·47,1·29)	1·69 (1·37,2·09)
Uganda	1·56 (1·32,1·85)	0·64 (0·41,1)	2·46 (2·05,2·95)	0·8 (0·52,1·23)	2·68 (2·29,3·13)
Zambia	3·78 (3·33,4·28)	1·82 (1·36,2·42)	5·7 (4·97,6·52)	2·19 (1·56,3·07)	4·47 (3·91,5·1)
Zimbabwe	3·75 (3·35,4·2)	2·72 (2·23,3·31)	4·77 (4·16,5·45)	2·89 (2·41,3·47)	4·96 (4·32,5·69)

Note: HIV prevalence is a measure of the proportion of the population currently infected with HIV. The units are percentages (%). The 95% confidence intervals are included in the parenthesis. The estimates for Total, Male, and Female are among all age groups. The estimates for each age group is among both sex. A few countries have 0 observations for age group 10 - 14 due to lack of information on early sex and are not included in analysis.

Table B.2: Incidence rate of HIV among people aged 15 - 24 years old from the data used.

Country	Both sex	Male	Female
Cameroon	0·347 (0·151,0·543)	0·072 (0,0·206)	0·637 (0·184,1·088)
Côte d'Ivoire	0·066 (0,0·174)	0·093 (0,0·273)	0·039 (0,0·155)
Eswatini	1·073 (0·485,1·658)	0·517 (0,1·123)	1·67 (0·643,2·686)
Ethiopia	0·015 (0,0·061)	0·03 (0,0·137)	0 (0,0·216)
Lesotho	0·778 (0·308,1·246)	0·125 (0,0·411)	1·49 (0·624,2·349)
Malawi	0·216 (0·021,0·411)	0·046 (0,0·187)	0·379 (0·04,0·717)
Namibia	0·509 (0·193,0·824)	0·028 (0,0·139)	0·989 (0·393,1·582)
Rwanda	0·056 (0,0·129)	0·049 (0,0·148)	0·062 (0,0·168)
Tanzania	0·072 (0,0·159)	0 (0,0·229)	0·145 (0,0·308)
Uganda	0·405 (0·21,0·601)	0·26 (0·032,0·487)	0·547 (0·243,0·851)
Zambia	0·506 (0·231,0·78)	0·078 (0,0·241)	0·939 (0·44,1·435)
Zimbabwe	0·301 (0·082,0·518)	0·144 (0,0·367)	0·458 (0·094,0·821)

Note: The units are percentages (%) per year. HIV incidence is the measure of new infections of HIV per year. The 95% confidence intervals are included in the parenthesis. 2 countries (Tanzania and Rwanda) have more than 93% data missing on indicators of early sex. They are all not included in analysis. Note that incidence estimates are based on a small number of recent infections. The data were not powered to estimate HIV incidence at the national level; therefore, these estimates should be interpreted with caution.

Table B.3: Prevalence of early sexual debut among people aged 15 - 24 years old.

Country	Total	Male	Female	10 to 17	18 to 24
Cameroon	23·3 (22·13,24·52)	24·74 (22·91,26·66)	21·96 (20·49,23·5)	15·28 (13·74,16·96)	29·98 (28·42,31·6)
Côte d'Ivoire	36·77 (34·74,38·85)	42·05 (38·79,45·39)	32·35 (29·9,34·91)	65·89 (60·62,70·8)	31·59 (29·46,33·8)
Eswatini	9·95 (8·71,11·35)	12·31 (10·32,14·63)	7·77 (6·35,9·48)	5·91 (4·58,7·58)	13·21 (11·33,15·36)
Ethiopia	16·59 (14·99,18·33)	13·18 (10·72,16·1)	19·18 (17·17,21·36)	66·6 (54·95,76·53)	14·49 (12·96,16·17)
Lesotho	38·13 (36,40·31)	52·03 (48·91,55·13)	20·25 (17·89,22·84)	76·75 (72·47,80·54)	28·54 (26·36,30·83)
Malawi	37·74 (35·92,39·61)	46·52 (43·7,49·37)	29·19 (26·96,31·53)	77·07 (72·61,80·99)	29·51 (27·68,31·41)
Namibia	27·43 (25·66,29·28)	34·72 (31·92,37·64)	20·56 (18·52,22·77)	31·43 (27·54,35·6)	26·1 (24·14,28·17)
Rwanda	28·32 (26·94,29·75)	36·41 (34·26,38·62)	20·2 (18·55,21·96)	85·4 (81·77,88·41)	22·22 (20·87,23·63)
Tanzania	34·7 (33·2,36·24)	38·98 (36·61,41·4)	30·77 (28·92,32·68)	72·19 (68·47,75·63)	27·61 (26·08,29·19)
Uganda	38·86 (37·51,40·24)	45·67 (43·59,47·77)	32·58 (30·85,34·36)	79·14 (76·43,81·61)	29·42 (28·11,30·77)
Zambia	37·5 (35·92,39·11)	43·27 (40·83,45·75)	31·84 (29·86,33·89)	74·91 (70·68,78·71)	31·23 (29·62,32·88)
Zimbabwe	10·89 (10·02,11·83)	13·2 (11·78,14·77)	8·65 (7·66,9·76)	10·51 (9·24,11·94)	11·23 (10·06,12·52)

Note: The estimates for Total, Male, and Female are among all age groups. The estimates for each age group is among both sex. A few countries have 0 observations for age group 10 - 14 due to lack of information on early sex and are not included.

Appendix C

STATISTICAL ANALYSIS

C.1 Covariates

The recency of an HIV infection is determined through a combination of tests[2, 3], including the Limiting Antigen Enzyme (LAg-Avidity) Immunoassay, viral load, and antiretroviral (ARV) test results[1]. Participants with blood test results indicative of a recent HIV infection are assigned a value of '1'. Conversely, those who are not HIV positive are designated a value of '0'[2].

This dichotomous outcome variable allows for a straightforward interpretation of the analysis results, as it directly represents the presence or absence of recent HIV infection among the participants. The assignment of these numerical values facilitates the use of statistical models, such as logistic regression, that are specifically designed to handle binary response variables.

The variable of early sexual debut is a dichotomous response (yes, no) to the question "Have you ever had sex?" for participants aged 10-14 years[3]. For those aged 15 years and

[1]The LAg-Avidity blood test detects recent HIV infection using a specially designed protein that binds to a wide range of HIV antibodies. It measures the strength of binding by staining strongly-bound antibodies, yielding a normalized optical density (ODn).

[2]As those who possess long-term infections are not at risk of developing new infections, they are dropped in the modeling

[3]In the questionnaire for under-15, the question "Do you know what sex is?" is first asked. If the answer is no, the questions related to sex will be skipped. If the answer is yes, a second question with definition will be asked: "Have you ever had vaginal, anal, or oral sex? Vaginal sex is when a penis enters a vagina. Anal sex is when a penis enters an anus. Oral sex is when a person puts his/her mouth on the penis or vagina of another person." If the answer is yes again, the question "How old were you when you had sex for the very first time?" will then be asked.

older, it is determined by the response to the question ”How old were you when you had sex for the very first time?”[4]. Specifically, it is defined as sexual initiation before the age of 18 years in this study. We choose this cut-off age based on the legal consideration. From a legal standpoint, the age of consent in many jurisdictions is set at 18[4], the threshold at which an individual is legally recognized as mature enough to give informed consent to sexual activity. In many places, 18 is the minimum age for obtaining a driver's license without restrictions or supervision[5]. Aligning our definition of early sexual debut with this legal benchmark ensures that our study adheres to widely accepted legal standards.

The age variable is a continuous measure ranging from 10 to 24 years. Sex is categorised into male or female, and the place of residence is divided into rural and urban areas.

The education level is dichotomised into the categories of having received any education or no education. This simplification is based on the assumption that the majority of individuals under 15 years are unlikely to have completed middle school.

The household wealth is represented by a categorical wealth quintile (1 - 5), derived from dwelling characteristics and asset variables. This classification follows the guidelines provided by the Demographic and Health Surveys (DHS)[6].

The country of residence is also included as a categorical variable. The covariates are carefully selected to provide a comprehensive understanding of the factors influencing the risk of HIV infection among both adolescents and young adults.

C.2 Data imputation

Some observations in the survey have missing data for some covariates. The primary reason of imputation of missing data is to mitigate the bias resulting from missingness, rather than discarding incomplete cases altogether. Studies[7] have shown that approximately 94%

[4]For over-15, there's an option of ”NEVER HAD SEX” for the question ”How old were you when you had sex for the very first time?”

of research that use listwise deletion to eliminate entire observation can result in loss of valuable information. For this research, we impute the missing data by employing the multiple imputation by chained equations (MICE). MICE is a statistical method widely used in health science to handle missing data in a dataset, and has been proven to outperform other imputation techniques in some simulation experiments[8, 9].

Multiple imputation involves creating multiple imputed datasets to account for the missing data and then applying standard analysis methods (hypothesis testing in our case) to each of these datasets. The multiple sets of results are combined using Rubin's rule[10] to get final estimates with standard errors that allow for the uncertainty of the missing data. Specifically, our parameter of interest would be the coefficient (β) of early sexual debut. We could obtain an estimate $\hat{\beta}_m$ with each of the M imputed dataset as well as their standard errors se_m. To get the final point estimate $\hat{\beta}$, we would average over the estimates from each imputed data; and to get the standard error se_β of the final estimate, we would combine the between-imputation variance and the within-imputation variance:

$$se_\beta = \sqrt{W + \left(1 + \frac{1}{M}\right) B}$$

where the within-imputation variance is estimated by $W = \frac{1}{M}\sum_{m=1}^{M} se_m^2$, and the between-imputation variance is estimated by $B = \frac{1}{M-1}\sum_{m=1}^{M}(\hat{\beta}_m - \hat{\beta})^2$.

The procedures and advantages of MICE are well documented[11]. We have followed the suggestions of using the outcome for imputation of missing predictor values proposed by several studies[12, 13]. We implement the imputation of missing data using the MICE mice package[14] in the R statistical software.

C.3 Modeling

The statistical analysis for this study involves the implementation of multivariate logistic regression models, adjusted to take into account the survey sampling design. These models are employed to examine the associations between early sexual debut and HIV infection among both adolescents and young adults. The models control for potential confounding variables, and incorporate a fixed effect to account for variability at the country level. We model the associations between new HIV infections and covariates among adolescents and young adults with the Eq. C.1 described below.

$$\begin{aligned} logit(y) = \beta_0 + \beta_1 \cdot \text{early\_sex} + \beta_2 \cdot \text{gender} + \beta_3 \cdot \text{wealth\_quintile} \\ + \beta_4 \cdot \text{educated} + \beta_5 \cdot \text{urban} + \beta_6 \cdot \text{age} + \beta_7 \cdot \text{country} + \epsilon \end{aligned} \tag{C.1}$$

Where the variables are described as follows,

- **Dependent Variable**:
  - y: Represents whether an individual has a recent infection (binary outcome).
- **Independent Variables**:
  - `early_sex`: A binary variable indicating whether the individual has had early sexual debut.
  - `gender`: A binary variable, with female as the reference category, representing the gender of the individual.
  - `wealth_quintile`: Categorical variable with values ranging from 1 to 5, characterizing the individual's household wealth.

- `educated`: Binary variable indicating whether the individual has received any education.
- `urban`: A binary variable indicating whether the individual lives in urban area.
- `age`: Continuous variable reflecting the age of the individual.
- `country`: Categorical variable denoting the individual's country of residence, with Zambia selected as the reference category (Zambia has the highest number of recent infections).

- **Parameters**:
  - $\beta_0, \beta_1, \ldots, \beta_7$: Coefficients representing the change in the log odds of having a recent infection for a one-unit change in the corresponding variable, holding other variables constant.
  - ϵ: Error term, capturing unobserved variability in the dependent variable.

Table C.1: Odds ratio (OR) and 95% confidence intervals (CI) from the baseline model.

**variable**	**OR**	**Lower bound**	**Upper bound**
Early Sex	2·65	1·50	4·73
Male	0·24	0·12	0·48
Wealthquintile	1·13	0·78	1·66
Educated	0·72	0·26	2·02
Urban	1·30	0·61	2·78
Age	1·32	1·23	1·40
Côte d'Ivoire	0·10	0·03	0·38
Ethiopia	0·03	0·00	0·19
Cameroon	0·64	0·25	1·61
Uganda	0·79	0·36	1·74
Malawi	0·48	0·18	1·26
Namibia	0·90	0·38	2·13
Zimbabwe	0·68	0·28	1·62
Eswatini	2·60	1·20	5·66
Lesotho	1·81	0·84	3·90
Tanzania	0·14	0·04	0·45

Note: The reference country for the country variable is Zambia.

Appendix D

SENSITIVITY ANALYSIS

The number of imputations performed in handling missing data might influence the study's results. There have been some debates regarding how many imputations are needed for good statistical inference. Some research suggest that 3 – 5 imputations are sufficient to yield excellent results[15]. Other studies show that the statistical power for small effect sizes diminishes as the number of imputations become smaller and recommend performing more imputations than previously considered sufficient[16, 17]. In our sensitivity analysis, we vary the sets of imputations to be 10, 20 and 50. Additionally, we conduct the model analysis without data imputations, and any observations containing missing data are excluded from the analysis (complete-case analysis).

Furthermore, the choice of imputation algorithm can significantly impact the results of a study. Several imputation algorithms exist, each with its strengths and weaknesses[18, 19], and the choice between them can alter the outcomes of the research. Therefore, a sensitivity analysis that examines the effects of different imputation algorithms will provide insights into how our findings might change under these different approaches. This will help ensure that our results are not inappropriately influenced by the particular imputation algorithm selected. In our sensitivity analysis, we have imputed the data using three other imputation algorithms: random sample from observed values, classification and regression trees[20], and Bayesian linear regression[21].

The decision to include adolescents as young as 10 to 14 years old in the study could have substantial implications for our findings. Given the sensitive and complex nature of

sexual behavior in this age group[22], their inclusion could introduce additional variability and potential bias into our results. Conducting a sensitivity analysis that compares results with and without this age group will allow us to assess the impact of this decision on our overall conclusions.

The definition of "early" sexual debut can vary, and the choice of a cutoff age can influence the study's outcomes. Some studies use 15 as the cutoff age, while others may use 16, 17, or 18[23, 24, 25]. In our sensitivity analysis, we explore the impact of varying the cutoff age for early sexual debut from 15 to 18 years. This will help us understand how sensitive our model is to the definition of "early" sexual debut.

Gender can play a significant role in sexual behavior and its associated outcomes. Research has shown that males and females often differ in their sexual behaviors, attitudes, and risks. For example, females tend to have much older sexual partner and forced sex compared to males[26, 27, 28, 29]. To account for potential gender differences, we conduct separate models for males and females in the sensitivity analysis. This will allow us to identify any gender-specific patterns or biases that may exist in our data.

The effect of early sexual debut might not be uniform across genders due to various factors, including biological differences and social norms. For example, females who experience early sexual debut may be at a higher risk for HIV because of biological vulnerability and societal factors[30, 31]. Therefore, we include an interaction term between gender and early sexual debut in our sensitivity analysis. By including this interaction term, we can provide a more comprehensive understanding of the impact of early sexual debut, taking into account the complex interplay between gender and early sexual debut.

Country-level factors, such as HIV prevalence, could confound the relationship between early sexual debut and our outcome variable. In our sensitivity analysis, we include country-level HIV prevalence rates as a covariate while dropping the country variable. This will

help us assess if country-level prevalence can account for the variations observed between countries.

The choice of imputation method can potentially influence the results of a study. To assess the robustness of our findings, we conduct a sensitivity analysis comparing two widely-used imputation methods for multiple imputation: MICE and Amelia[32]. Their usefulness may vary depending on the missing data mechanism and the underlying distribution of the data. By comparing the results obtained using these two different imputation methods, we aim to better understand the impact of early sexual debut on recent HIV infections.

Lastly, censoring can introduce bias and affect the validity of study findings. In our sensitivity analysis, we investigate the impact of dropping observations that are below the cutoff age for early sexual debut because the exposure of interest (early sexual debut) is not observed for these subjects in the study. This will help us assess how sensitive our model is to incomplete data.

In summary, by conducting a sensitivity analysis on these nine hyperparameters, we can gain a more comprehensive and robust understanding of the relationship between early sexual debut and the risk of HIV infection among adolescents and young adults. This will enhance the validity and overall quality of our research findings. In the process of sensitivity analysis, we only investigate one parameter at a time while keeping the other parameters constant. We compare the obtained results with those from the benchmark model.

D.0.1 Number of data imputations

Figure D.1 displays the sensitivity analysis that compares the model results when varying the number of data imputations before modeling. Note that we have used 5 sets of data imputations in our benchmark model. On the X-axis, we have the odds ratios for different covariates. The color of each line represents a different set of imputations. For each covariate,

we see that the odds ratios slightly fluctuate as the number of imputations changes. However, the statistical significance at the 95% confidence level remain the same for all covariates. Therefore, our model's conclusions regarding all covariates are largely robust to the number of data imputations. The data behind the figure are shown in D.1.

Figure D.1: Sensitivity analysis comparing the model results of varying the number of data imputations.

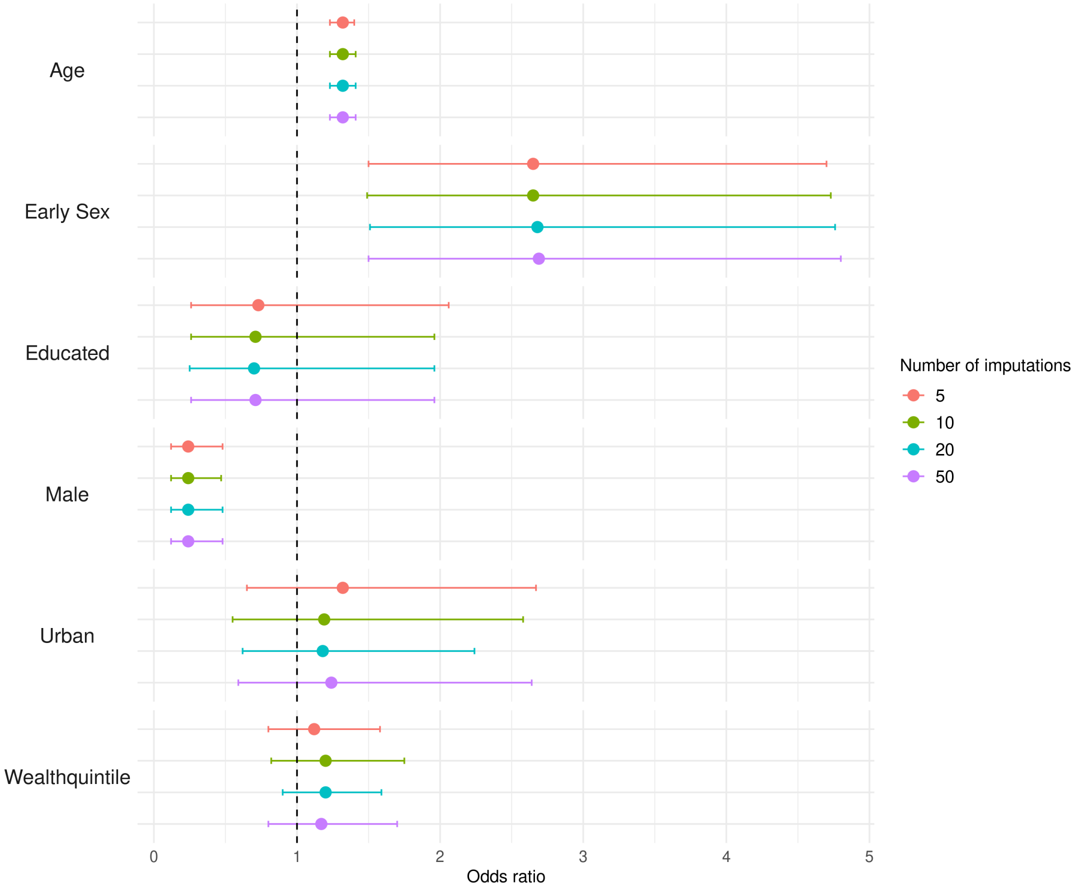

Note: The odds ratios are associated with one-unit increase in the covariate relative to the reference group, holding all other variables constant. The benchmark model have used 5 sets of data imputation. The dashed line indicates an odds ratio of 1.

D.0.2 Imputation algorithms

Figure D.2 shows the sensitivity analysis that compares the data imputation models. In our benchmark model we have used predictive mean matching (pmm) for data imputation. Similar to the sensitivity analysis comparing the number of data imputations, the odds ratios change slightly as the imputation algorithms change, but the conclusions regarding all covariates still hold. The data for the figure are shown in D.2.

D.0.3 Whether to include young adolescents

The sensitivity analysis that compares the model results of whether to include young adolescents in the analysis is shown in Figure D.3. Note that we have included young adolescents in our benchmark model. The graph shows that the conclusions with respect to all covariates remain the same whether to include young adolescents or not. The data are shown in D.3.

D.0.4 Cutoff age for early sexual debut

The sensitivity analysis that compares the model results of using different cutoff age to classify early sexual debut in the analysis is shown in Figure D.4. Note that we have used 18 years old as cutoff age in our benchmark model. The graph shows that the conclusions with respect to all covariates remain the same for cutoff age from 16 to 18 years old. The effect of sexual debut slightly decreases as the cutoff age gets younger. The results are shown in D.4.

D.0.5 Gender-specific models

The sensitivity analysis that compares the model results of whether to model males and females separately is shown in Figure D.5. Note that we have modeled them together in the benchmark model. The graph shows that the effect of early sexual debut is significant for females and not significant for the males. The uncertainty interval is much wider for males

Figure D.2: Sensitivity analysis comparing the model results of using different data imputation algorithms.

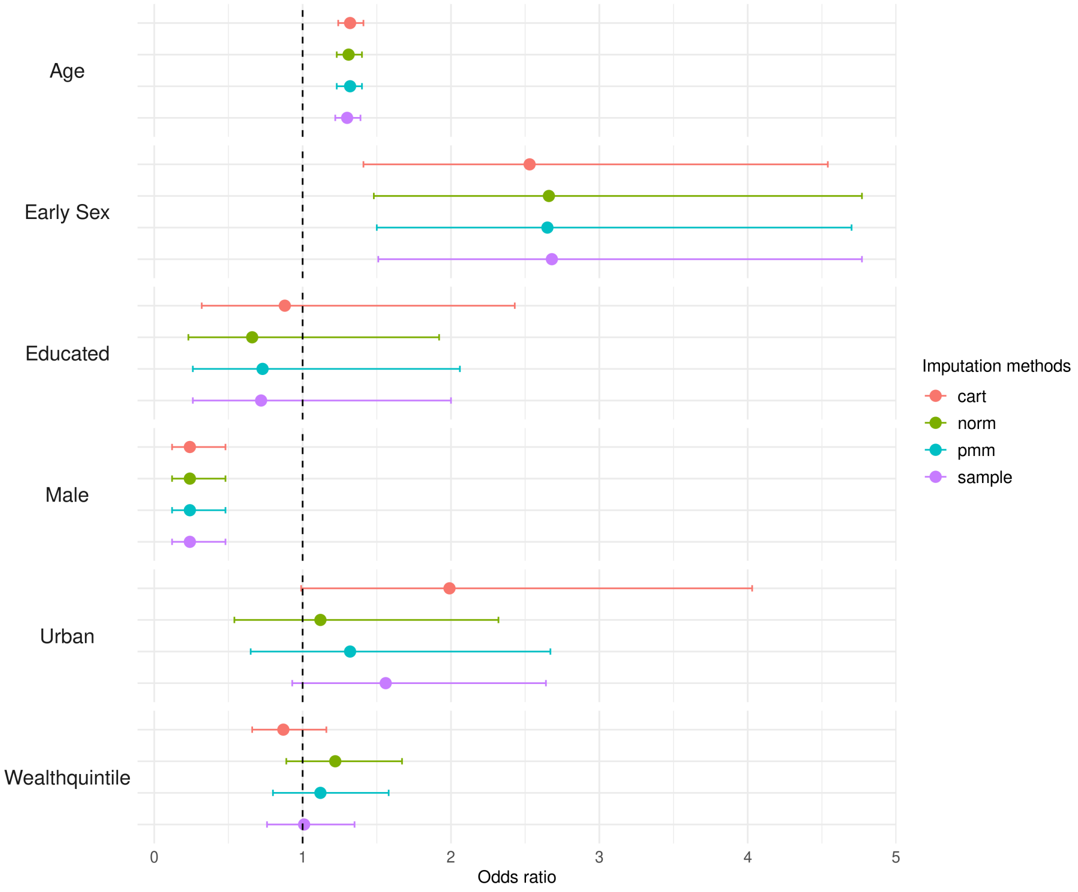

Note: The odds ratios are associated with one-unit increase in the covariate relative to the reference group, holding all other variables constant. cart: classification and regression trees. norm: Bayesian linear regression. pmm: predictive mean matching. sample: random sample from observed values. The benchmark model have used predictive mean matching for data imputation.

Figure D.3: Sensitivity analysis comparing the model results of whether to include young adolescents (10 - 14 years).

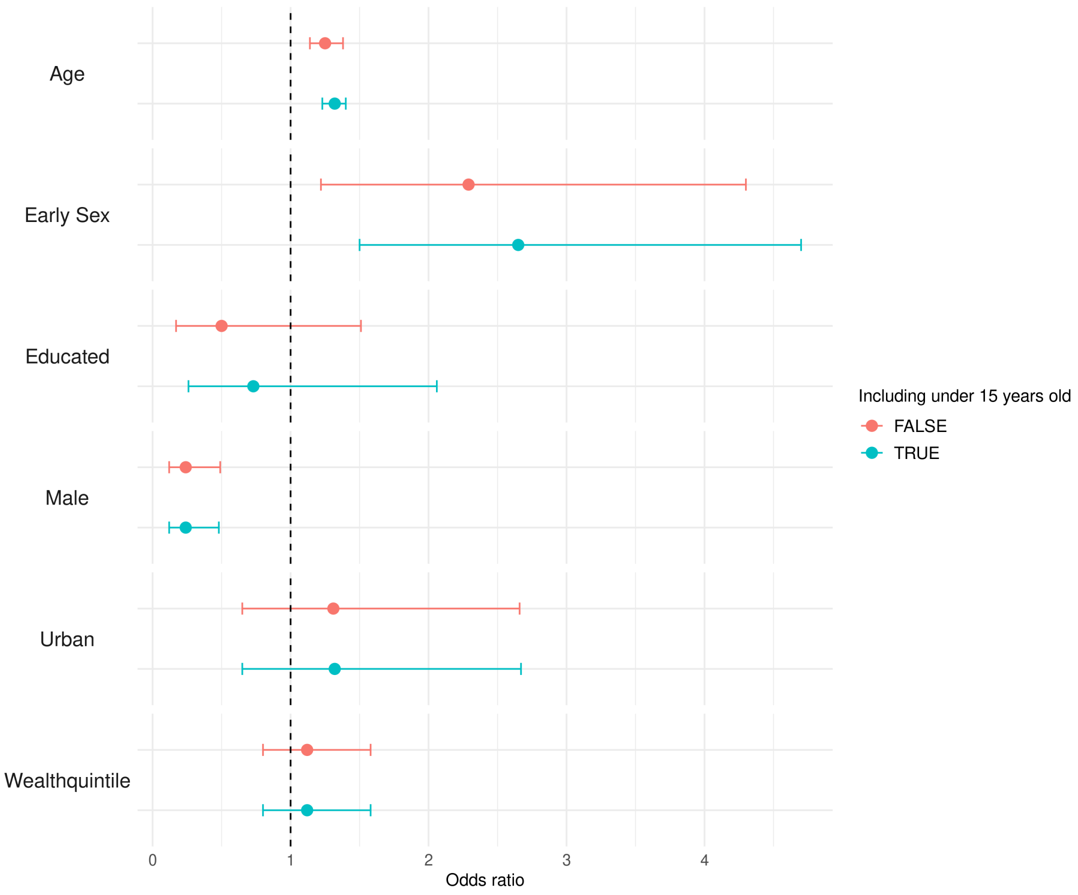

Note: The odds ratios are associated with one-unit increase in the covariate relative to the reference group, holding all other variables constant. The benchmark model have included young adolescents for analysis.

Figure D.4: Sensitivity analysis comparing the model results of using different cutoff age to classify early sexual debut.

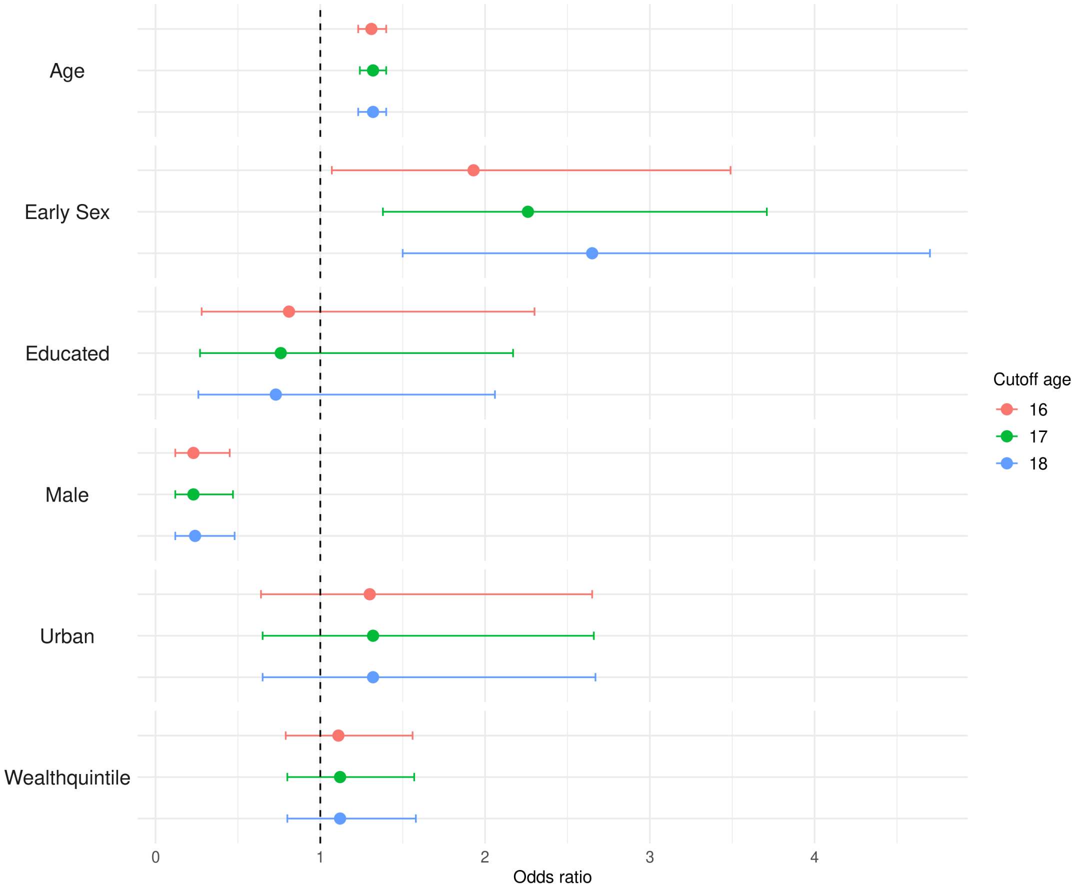

than for females, due to the smaller sample size for males after modeling them separately. This reduced statistical power might explain the lack of significance in the males. The data are shown in D.7.

Figure D.5: Sensitivity analysis comparing the model results of whether to model males and females separately.

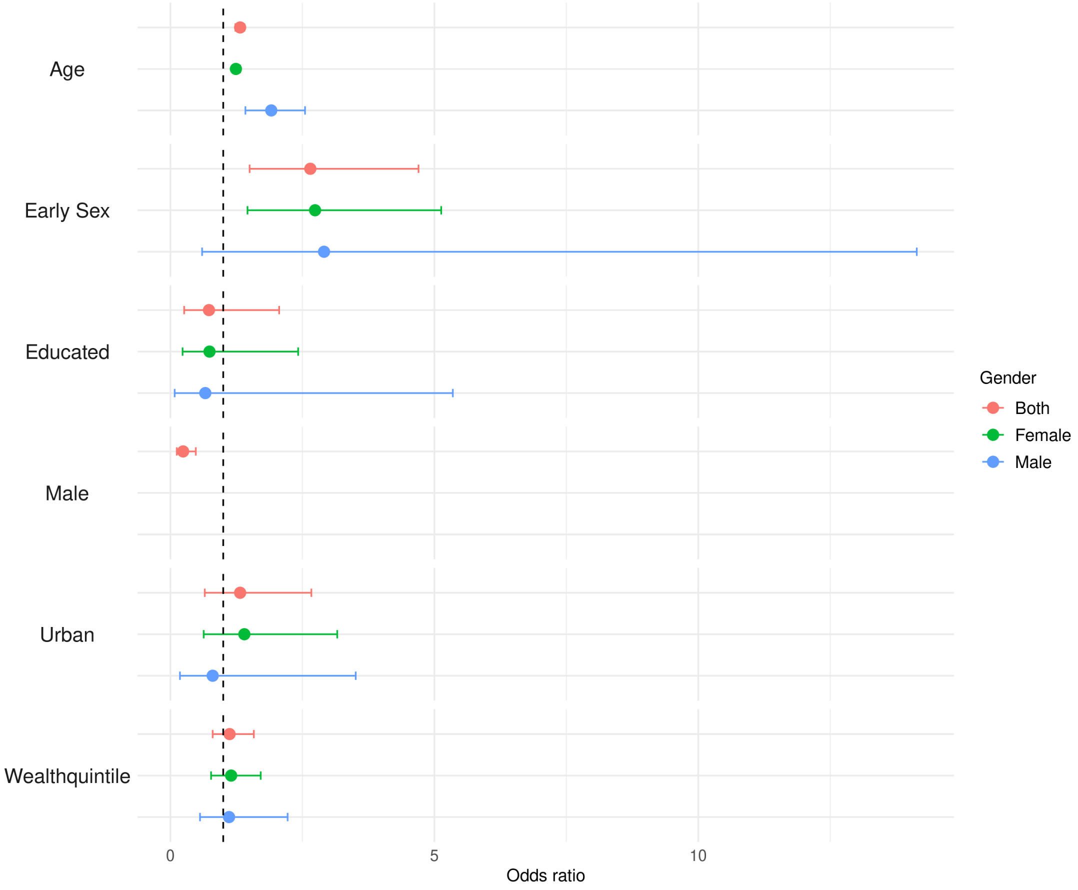

D.0.6 Interaction between Gender and Early Sexual Debut

The sensitivity analysis that compares the model results of whether to include the interaction between gender and early sexual debut in the model is shown in Figure D.6. Note that we have excluded the interaction in our benchmark model. The graph shows that the conclusions

with respect to all covariates remain the same whether to include the interaction or not. The data are shown in D.8. Note that the effects of gender and the interaction are not significant after including the interaction. This suggests that the effect of early sexual debut on the outcome might not depend on the gender.

Figure D.6: Odds ratios from the models of sensitivity analysis of interaction between early sex and gender.

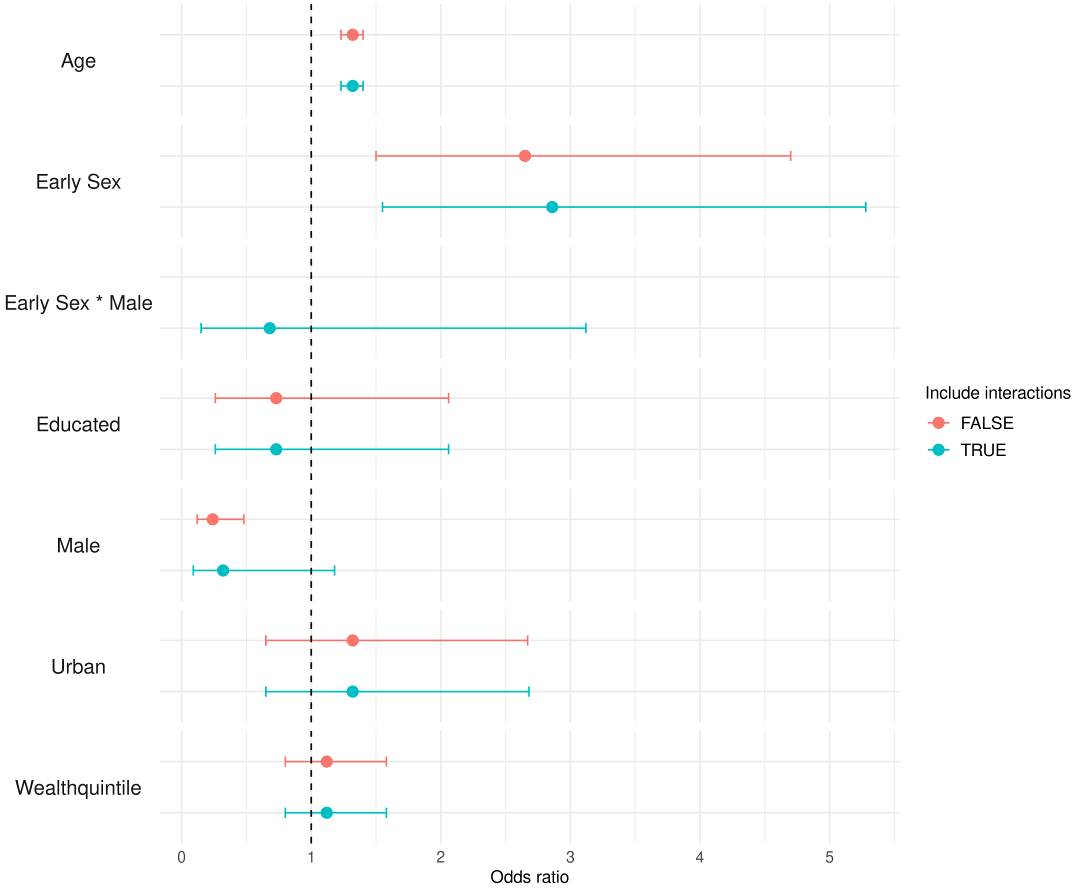

D.0.7 Country-level HIV prevalence as covariate

The sensitivity analysis that compares the model results of whether to include national HIV prevalence as covariate in the model is shown in Figure D.7. Note that we have not included

the prevalence in our benchmark model. The graph shows that the conclusions with respect to all covariates remain the same whether to include the HIV prevalence or not. The data are shown in D.5. Note that in order to include HIV prevalence as covariate, we need to drop the country variable since they are collinear. A unit increase in the prevalence is associated with 10% increase in the odds ratio of getting infected with HIV (95% CI: [7%, 13%]).

Figure D.7: Sensitivity analysis comparing the model results of whether to include HIV prevalence as covariate.

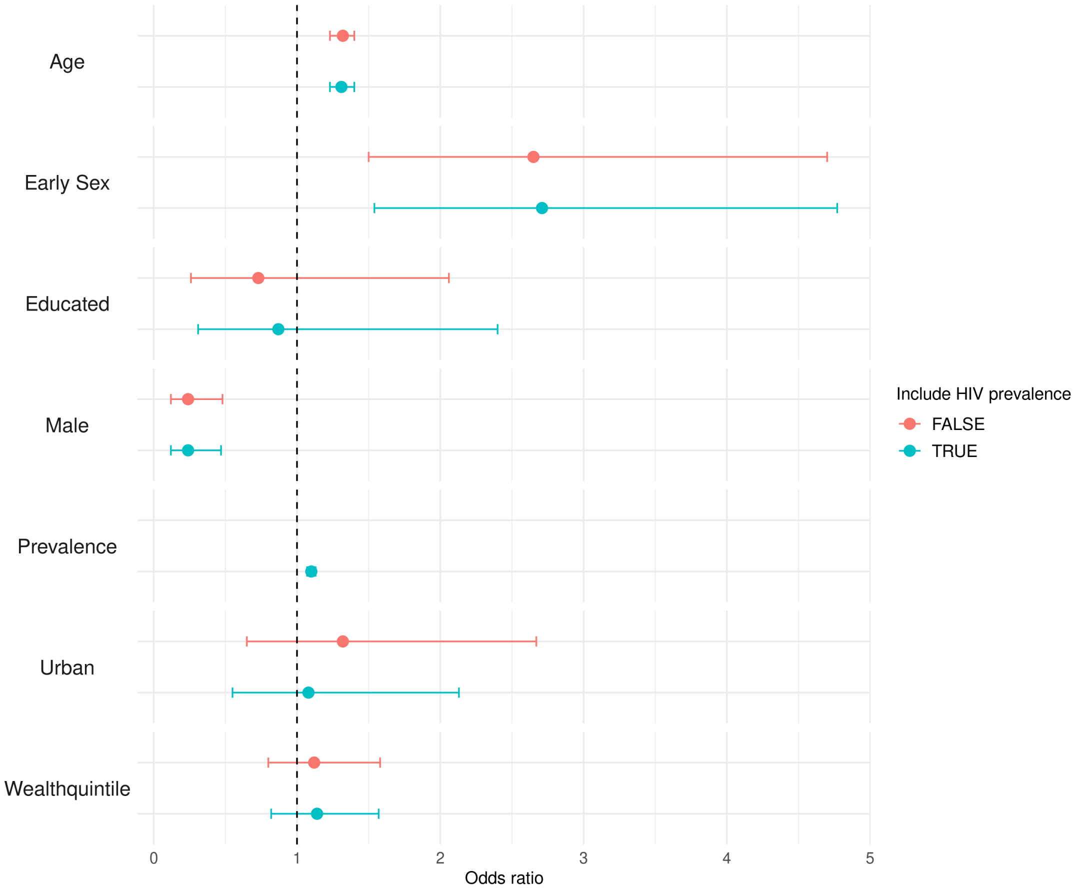

D.0.8 Handling censoring

The sensitivity analysis that compares the model results of whether to drop the censored data in the analysis is shown in Figure D.8. We have not dropped censored data in the benchmark model. The graph shows that the conclusions with respect to all covariates remain the same whether to drop the censored data. The data are shown in D.6.

Figure D.8: Sensitivity analysis comparing the model results of handling censored data.

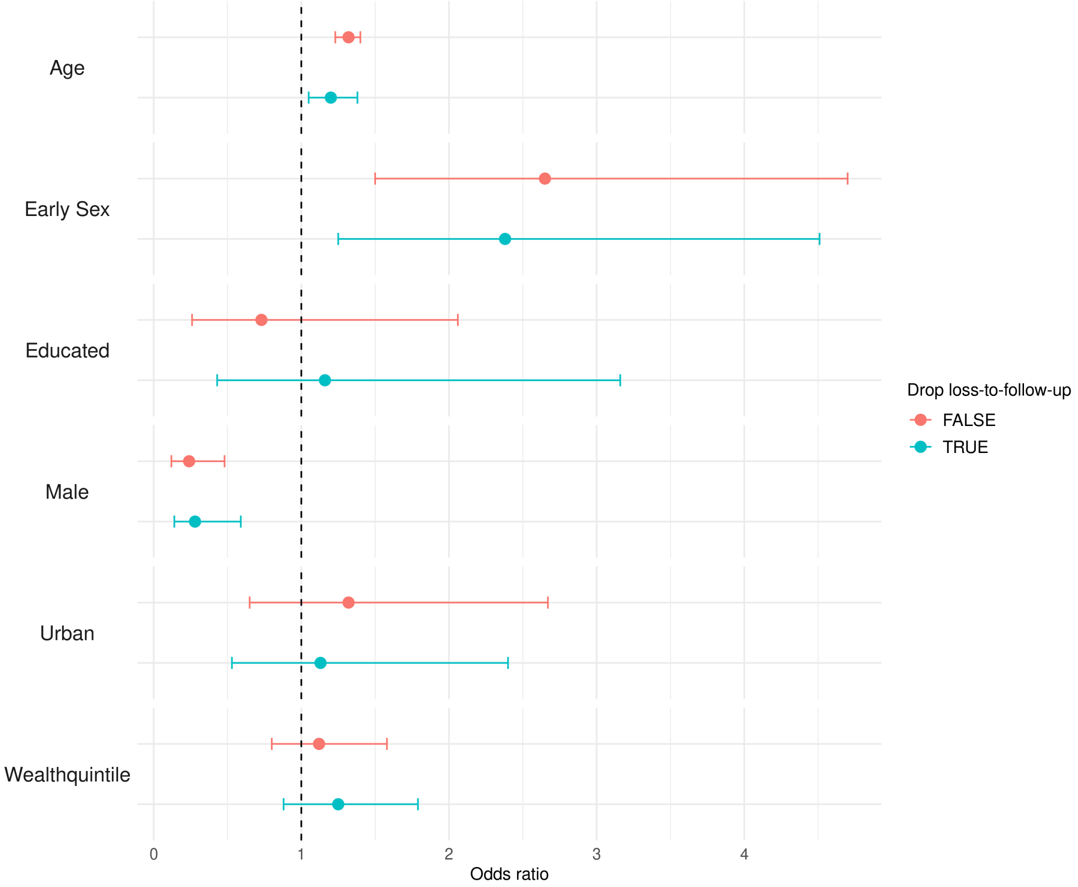

Note: If the cutoff age is 18 for classifying early sexual debut, exclude censored data means that observations below 18 are dropped.

D.0.9 Imputation method

The sensitivity analysis that compares the model results of using Amelia or MICE to impute the data before the analysis is shown in Figure D.9. We have used MICE to impute the missing data in the benchmark model. The graph shows that there is only slight difference in the results and the conclusions with respect to all covariates hold whether to use Amelia or MICE for imputation. The data are shown in D.9.

Figure D.9: Sensitivity analysis comparing the model results of imputation methods.

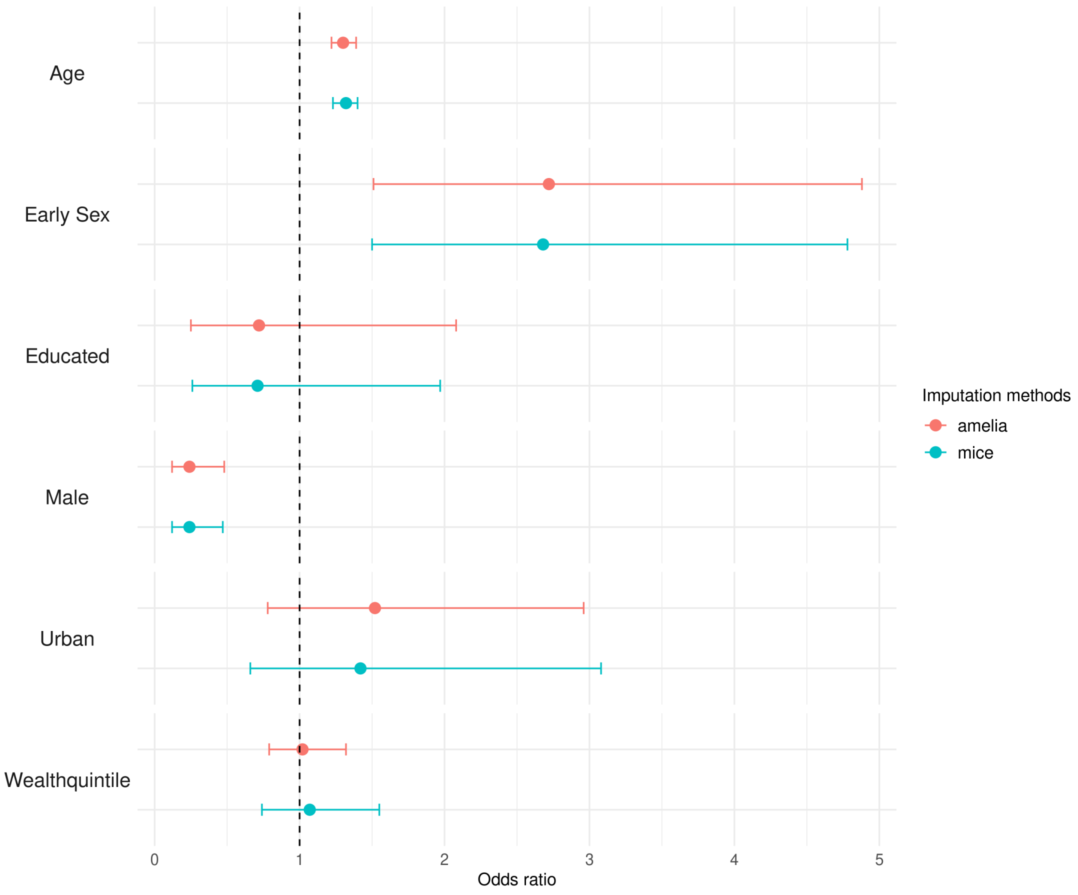

Table D.1: Odds ratios from the models of sensitivity analysis of number of data imputations.

variable	OR (No imputation)	OR (5 sets)	OR (10 sets)	OR (20 sets)	OR (50 sets)
Early Sex	2.89 (0.96, 8.69)	2.64 (1.49, 4.68)	2.6 (1.45, 4.65)	2.64 (1.48, 4.7)	2.64 (1.49, 4.67)
Age	1.04 (0.95, 1.13)	1.31 (1.22, 1.39)	1.3 (1.22, 1.39)	1.3 (1.22, 1.39)	1.3 (1.22, 1.39)
Educated	0.69 (0.1, 4.8)	0.72 (0.25, 2.01)	0.73 (0.26, 2.05)	0.71 (0.26, 1.96)	0.71 (0.26, 1.97)
Male	0.02 (0, 0.08)	0.25 (0.12, 0.5)	0.25 (0.12, 0.5)	0.25 (0.12, 0.5)	0.25 (0.12, 0.5)
Urban	1.6 (0.42, 6.13)	1.16 (0.54, 2.5)	1.41 (0.6, 3.31)	1.32 (0.64, 2.74)	1.27 (0.61, 2.64)
Wealthquintile	1.52 (1.05, 2.19)	1.21 (0.8, 1.83)	1.07 (0.69, 1.66)	1.11 (0.79, 1.57)	1.14 (0.8, 1.62)

Table D.2: Odds ratios from the models of sensitivity analysis of imputation methods.

variable	OR (cart)	OR (norm)	OR (pmm)	OR (sample)
Early Sex	2.53 (1.41, 4.54)	2.66 (1.48, 4.77)	2.65 (1.5, 4.7)	2.68 (1.51, 4.77)
Age	1.32 (1.24, 1.41)	1.31 (1.23, 1.4)	1.32 (1.23, 1.4)	1.3 (1.22, 1.39)
Educated	0.88 (0.32, 2.43)	0.66 (0.23, 1.92)	0.73 (0.26, 2.06)	0.72 (0.26, 2)
Male	0.24 (0.12, 0.48)	0.24 (0.12, 0.48)	0.24 (0.12, 0.48)	0.24 (0.12, 0.48)
Urban	1.99 (0.99, 4.03)	1.12 (0.54, 2.32)	1.32 (0.65, 2.67)	1.56 (0.93, 2.64)
Wealthquintile	0.87 (0.66, 1.16)	1.22 (0.89, 1.67)	1.12 (0.8, 1.58)	1.01 (0.76, 1.35)

Note: cart: classification and regression trees. norm: Bayesian linear regression. pmm: predictive mean matching. sample: random sample from observed values.

Table D.3: Odds ratios from the models of sensitivity analysis of whether to include data of young adolescents.

variable	OR (Excluding under 15)	OR (Including under 15)
Early Sex	2.29 (1.22, 4.3)	2.65 (1.5, 4.7)
Age	1.25 (1.14, 1.38)	1.32 (1.23, 1.4)
Educated	0.5 (0.17, 1.51)	0.73 (0.26, 2.06)
Male	0.24 (0.12, 0.49)	0.24 (0.12, 0.48)
Urban	1.31 (0.65, 2.66)	1.32 (0.65, 2.67)
Wealthquintile	1.12 (0.8, 1.58)	1.12 (0.8, 1.58)

Table D.4: Odds ratios from the models of sensitivity analysis of the cutoff age for early sexual debut.

**variable**	**OR (16 years)**	**OR (17 years)**	**OR (18 years)**
Early Sex	1·93 (1·07, 3·49)	2·26 (1·38, 3·71)	2·65 (1·5, 4·7)
Age	1·31 (1·23, 1·4)	1·32 (1·24, 1·4)	1·32 (1·23, 1·4)
Educated	0·81 (0·28, 2·3)	0·76 (0·27, 2·17)	0·73 (0·26, 2·06)
Male	0·23 (0·12, 0·45)	0·23 (0·12, 0·47)	0·24 (0·12, 0·48)
Urban	1·3 (0·64, 2·65)	1·32 (0·65, 2·66)	1·32 (0·65, 2·67)
Wealthquintile	1·11 (0·79, 1·56)	1·12 (0·8, 1·57)	1·12 (0·8, 1·58)

Table D.5: Odds ratios from the models of sensitivity analysis of whether to include HIV prevalence as covariate.

**variable**	**OR (Exclude HIV prevalence)**	**OR (Include HIV prevalence)**
Early Sex	2.65 (1.5, 4.7)	2.71 (1.54, 4.77)
Age	1.32 (1.23, 1.4)	1.31 (1.23, 1.4)
Educated	0.73 (0.26, 2.06)	0.87 (0.31, 2.4)
Male	0.24 (0.12, 0.48)	0.24 (0.12, 0.47)
Prevalence	NA	1.1 (1.07, 1.13)
Urban	1.32 (0.65, 2.67)	1.08 (0.55, 2.13)
Wealthquintile	1.12 (0.8, 1.58)	1.14 (0.82, 1.57)

Note: To include HIV prevalence as covariate, the country variable need to be dropped due to collinearity.

Table D.6: Odds ratios from the models of sensitivity analysis of handling censored data.

**variable**	**OR (Include censored data)**	**OR (Exclude censored data)**
Early Sex	2.65 (1.5, 4.7)	2.38 (1.25, 4.51)
Age	1.32 (1.23, 1.4)	1.2 (1.05, 1.38)
Educated	0.73 (0.26, 2.06)	1.16 (0.43, 3.16)
Male	0.24 (0.12, 0.48)	0.28 (0.14, 0.59)
Urban	1.32 (0.65, 2.67)	1.13 (0.53, 2.4)
Wealthquintile	1.12 (0.8, 1.58)	1.25 (0.88, 1.79)

Note: If the cutoff age is 18 for classifying early sexual debut, exclude censored data means that observations below 18 are dropped.

Table D.7: Odds ratios from the models of sensitivity analysis of whether to model gender separately.

variable	OR (Both)	OR (Female model)	OR (Male model)
Early Sex	2.65 (1.5, 4.7)	2.74 (1.46, 5.13)	2.91 (0.6, 14.14)
Age	1.32 (1.23, 1.4)	1.24 (1.17, 1.32)	1.91 (1.42, 2.55)
Educated	0.73 (0.26, 2.06)	0.74 (0.23, 2.42)	0.66 (0.08, 5.35)
Male	0.24 (0.12, 0.48)	NA	NA
Urban	1.32 (0.65, 2.67)	1.4 (0.63, 3.16)	0.8 (0.18, 3.51)
Wealthquintile	1.12 (0.8, 1.58)	1.15 (0.77, 1.71)	1.11 (0.56, 2.22)

Table D.8: Odds ratios from the models of sensitivity analysis of interaction between early sex and gender.

variable	OR (Excluding interaction)	OR (Including interaction)
Early Sex	2.65 (1.5, 4.7)	2.86 (1.55, 5.28)
Age	1.32 (1.23, 1.4)	1.32 (1.23, 1.4)
Early Sex * Male	NA	0.68 (0.15, 3.12)
Educated	0.73 (0.26, 2.06)	0.73 (0.26, 2.06)
Male	0.24 (0.12, 0.48)	0.32 (0.09, 1.18)
Urban	1.32 (0.65, 2.67)	1.32 (0.65, 2.68)
Wealthquintile	1.12 (0.8, 1.58)	1.12 (0.8, 1.58)

Table D.9: Odds ratios from the models of sensitivity analysis of imputation methods.

variable	OR (Amelia)	OR (MICE)
Early Sex	2.72 (1.51, 4.88)	2.68 (1.5, 4.78)
Age	1.3 (1.22, 1.39)	1.32 (1.23, 1.4)
Educated	0.72 (0.25, 2.08)	0.71 (0.26, 1.97)
Male	0.24 (0.12, 0.48)	0.24 (0.12, 0.47)
Urban	1.52 (0.78, 2.96)	1.42 (0.66, 3.08)
Wealthquintile	1.02 (0.79, 1.32)	1.07 (0.74, 1.55)

Appendix E

POPULATION ATTRIBUTABLE FRACTIONS

In the study, we calculate the PAF to quantify the proportion of HIV infections that could be prevented if early sexual debut were eliminated in the age group of 10-24 years for both males and females across different countries. Specifically, we estimate the PAF using the following formula,

$$PAF = \frac{Prev \cdot (OR - 1)}{1 + Prev \cdot (OR - 1)}$$

where $Prev$ is the prevalence of early sexual debut in each country. OR is the odds ratio of early sexual debut from the model as shown in Table C.1.

To account for the uncertainties in the model, we have integrated the variability of both the prevalence of early sexual debut and the odds ratio associated with early sexual debut into our estimates of the PAF. We employ a Monte Carlo simulation approach to generate the robust estimates. Specifically, both the prevalence and the odds ratio of early sexual debut are simulated 1000 times, drawing from distributions defined by their respective confidence intervals. The simulation allows us to incorporate the full range of plausible values for these variables, thereby providing a more comprehensive view of the potential impact of early sexual debut on HIV infections.

The simulated values for prevalence and odds ratio are then used as inputs in the formula for calculating PAF. By propagating these uncertainties through to the final estimates, we are able to calculate not only a point estimate for PAF but also a confidence interval that

Table E.1: PAF for male aged 10 - 24 years in each country.

Country	Estimate	Lower	Upper
Eswatini	17.4	5.7	32.9
Ethiopia	18.4	6.3	33.7
Zimbabwe	18.4	6.3	33.7
Cameroon	29.2	11.2	48.5
Namibia	36.3	15.0	57.1
Rwanda	37.4	15.6	58.3
Tanzania	38.9	16.7	59.7
Côte d'Ivoire	40.6	17.9	61.5
Zambia	41.3	18.0	62.0
Uganda	42.5	18.6	63.6
Malawi	43.0	19.0	63.7
Lesotho	45.6	20.5	66.4

reflects the combined uncertainties of both the prevalence and the odds ratio of early sexual debut.

Table E.2: PAF for female aged 10 - 24 years in each country.

Country	Estimate	Lower	Upper
Eswatini	11.8	3.6	23.6
Zimbabwe	13.0	4.2	24.8
Ethiopia	24.5	8.9	42.4
Rwanda	25.4	9.3	43.2
Lesotho	25.4	9.5	43.7
Namibia	25.7	9.6	43.9
Cameroon	26.9	10.2	45.6
Malawi	32.6	13.1	52.6
Tanzania	33.7	13.5	53.6
Zambia	34.4	13.8	54.7
Côte d'Ivoire	34.8	14.1	55.3
Uganda	34.9	13.9	55.2

References

[1] PHIA Collaborating institutions. *PHIA Project - Guiding the Global HIV Response*. PHIA Project. 2022. URL: https://phia.icap.columbia.edu/ (visited on 11/16/2022).

[2] Xierong Wei et al. "Development of Two Avidity-Based Assays to Detect Recent HIV Type 1 Seroconversion Using a Multisubtype Gp41 Recombinant Protein". In: *AIDS research and human retroviruses* 26.1 (Jan. 2010), pp. 61–71. ISSN: 1931-8405. DOI: 10.1089/aid.2009.0133. pmid: 20063992.

[3] Yen T. Duong et al. "Detection of Recent HIV-1 Infection Using a New Limiting-Antigen Avidity Assay: Potential for HIV-1 Incidence Estimates and Avidity Maturation Studies". In: *PloS One* 7.3 (2012), e33328. ISSN: 1932-6203. DOI: 10.1371/journal.pone.0033328. pmid: 22479384.

[4] *Age of Majority Law and Legal Definition | USLegal, Inc.* URL: https://definitions.uslegal.com/a/age-of-majority/ (visited on 07/25/2023).

[5] A. F. Williams. “Earning a Driver’s License”. In: *Public Health Reports (Washington, D.C.: 1974)* 112.6 (1997), pp. 452–461. ISSN: 0033-3549. pmid: 10822470.

[6] Shea Oscar Rutstein and Kiersten Johnson. *The DHS Wealth Index*. Calverton, Maryland: ORC Macro, 2004.

[7] Gary King et al. “Analyzing Incomplete Political Science Data: An Alternative Algorithm for Multiple Imputation”. In: *American Political Science Review* 95.1 (Mar. 2001), pp. 49–69. ISSN: 0003-0554, 1537-5943. DOI: 10.1017/S0003055401000235.

[8] Kimseth Seu, Mi-Sun Kang, and HwaMin Lee. “An Intelligent Missing Data Imputation Techniques: A Review”. In: *JOIV : International Journal on Informatics Visualization* 6.1-2 (May 31, 2022), p. 278. ISSN: 2549-9904, 2549-9610. DOI: 10.30630/joiv.6.1-2.935.

[9] Mohaimanul Hoque Chowdhury, Muhammad Kamrul Islam, and Shahidul Islam Khan. “Imputation of Missing Healthcare Data”. In: *2017 20th International Conference of Computer and Information Technology (ICCIT)*. 2017 20th International Conference of Computer and Information Technology (ICCIT). Dhaka: IEEE, Dec. 2017, pp. 1–6. ISBN: 978-1-5386-1150-0. DOI: 10.1109/ICCITECHN.2017.8281805.

[10] Donald B. Rubin, ed. *Multiple Imputation for Nonresponse in Surveys*. Wiley Series in Probability and Statistics. Hoboken, NJ, USA: John Wiley & Sons, Inc., June 9, 1987. ISBN: 978-0-470-31669-6 978-0-471-08705-2. DOI: 10.1002/9780470316696.

[11] Melissa J. Azur et al. “Multiple Imputation by Chained Equations: What Is It and How Does It Work?” In: *International Journal of Methods in Psychiatric Research* 20.1 (Feb. 24, 2011), pp. 40–49. ISSN: 1049-8931. DOI: 10.1002/mpr.329. pmid: 21499542.

[12] Karel G. M. Moons et al. “Using the Outcome for Imputation of Missing Predictor Values Was Preferred”. In: *Journal of Clinical Epidemiology* 59.10 (Oct. 2006), pp. 1092–1101. ISSN: 0895-4356. DOI: 10.1016/j.jclinepi.2006.01.009. pmid: 16980150.

[13] Jonathan A. C. Sterne et al. “Multiple Imputation for Missing Data in Epidemiological and Clinical Research: Potential and Pitfalls”. In: *BMJ* 338 (June 29, 2009), b2393. ISSN: 0959-8138, 1468-5833. DOI: 10.1136/bmj.b2393. pmid: 19564179.

[14] Stef van Buuren and Karin Groothuis-Oudshoorn. “Mice: Multivariate Imputation by Chained Equations in R”. In: *Journal of Statistical Software* 45 (Dec. 12, 2011), pp. 1–67. ISSN: 1548-7660. DOI: 10.18637/jss.v045.i03.

[15] Joseph L. Schafer and Maren K. Olsen. “Multiple Imputation for Multivariate Missing-Data Problems: A Data Analyst’s Perspective”. In: *Multivariate Behavioral Research* 33.4 (1998), pp. 545–571. ISSN: 1532-7906. DOI: 10.1207/s15327906mbr3304_5.

[16] John W. Graham, Allison E. Olchowski, and Tamika D. Gilreath. “How Many Imputations Are Really Needed? Some Practical Clarifications of Multiple Imputation Theory”. In: *Prevention Science* 8.3 (Sept. 1, 2007), pp. 206–213. ISSN: 1573-6695. DOI: 10.1007/s11121-007-0070-9.

[17] Ian R. White, Patrick Royston, and Angela M. Wood. “Multiple Imputation Using Chained Equations: Issues and Guidance for Practice”. In: *Statistics in Medicine* 30.4 (2011), pp. 377–399. ISSN: 1097-0258. DOI: 10.1002/sim.4067.

[18] Anil Jadhav, Dhanya Pramod, and Krishnan Ramanathan. “Comparison of Performance of Data Imputation Methods for Numeric Dataset”. In: *Applied Artificial Intelligence* 33.10 (Aug. 24, 2019), pp. 913–933. ISSN: 0883-9514, 1087-6545. DOI: 10.1080/08839514.2019.1637138.

[19] Geeta Chhabra et al. "A Comparison of Multiple Imputation Methods for Data with Missing Values". In: *Indian Journal of Science and Technology* 10.19 (June 29, 2017), pp. 1–7. ISSN: 0974-5645, 0974-6846. DOI: 10.17485/ijst/2017/v10i19/110646.

[20] Leo Breiman et al. *Classification And Regression Trees*. 1st ed. Routledge, Oct. 19, 2017. ISBN: 978-1-315-13947-0. DOI: 10.1201/9781315139470.

[21] Ismaël Castillo, Johannes Schmidt-Hieber, and Aad Van Der Vaart. "Bayesian Linear Regression with Sparse Priors". In: *The Annals of Statistics* 43.5 (Oct. 1, 2015). ISSN: 0090-5364. DOI: 10.1214/15-AOS1334.

[22] Jeanne Brooks-Gunn and Frank F. Furstenberg. "Adolescent Sexual Behavior." In: *American Psychologist* 44.2 (1989), pp. 249–257. ISSN: 1935-990X, 0003-066X. DOI: 10.1037/0003-066X.44.2.249.

[23] B. Auvert et al. "Ecological and Individual Level Analysis of Risk Factors for HIV Infection in Four Urban Populations in Sub-Saharan Africa with Different Levels of HIV Infection". In: *AIDS (London, England)* 15 Suppl 4 (Aug. 2001), S15–30. ISSN: 0269-9370. DOI: 10.1097/00002030-200108004-00003. pmid: 11686462.

[24] Sue Napierala Mavedzenge et al. "Determinants of Differential HIV Incidence among Women in Three Southern African Locations". In: *Journal of Acquired Immune Deficiency Syndromes (1999)* 58.1 (Sept. 1, 2011), pp. 89–99. ISSN: 1944-7884. DOI: 10.1097/QAI.0b013e3182254038. pmid: 21654502.

[25] C. Boileau et al. "Sexual and Marital Trajectories and HIV Infection among Ever-Married Women in Rural Malawi". In: *Sexually Transmitted Infections* 85 Suppl 1 (Suppl_1 Apr. 2009), pp. i27–33. ISSN: 1472-3263. DOI: 10.1136/sti.2008.033969. pmid: 19307337.

[26] Audrey E. Pettifor et al. “Early Age of First Sex: A Risk Factor for HIV Infection among Women in Zimbabwe”. In: *AIDS* 18.10 (July 2, 2004), pp. 1435–1442. ISSN: 0269-9370. DOI: 10.1097/01.aids.0000131338.61042.b8.

[27] Christine E. Kaestle et al. “Young Age at First Sexual Intercourse and Sexually Transmitted Infections in Adolescents and Young Adults”. In: *American Journal of Epidemiology* 161.8 (Apr. 15, 2005), pp. 774–780. ISSN: 0002-9262. DOI: 10.1093/aje/kwi095.

[28] Simon Gregson et al. “HIV Decline Associated with Behavior Change in Eastern Zimbabwe”. In: *Science* 311.5761 (Feb. 3, 2006), pp. 664–666. DOI: 10.1126/science.1121054.

[29] Audrey Pettifor et al. “Early Coital Debut and Associated HIV Risk Factors among Young Women and Men in South Africa”. In: *International Perspectives on Sexual and Reproductive Health* 35.2 (June 2009), pp. 82–90. ISSN: 1944-0391. DOI: 10.1363/ifpp.35.082.09. pmid: 19620092.

[30] Quarraisha Abdool Karim, Sengeziwe Sibeko, and Cheryl Baxter. “Preventing HIV Infection in Women: A Global Health Imperative”. In: *Clinical Infectious Diseases: An Official Publication of the Infectious Diseases Society of America* 50 Suppl 3 (Suppl 3 May 15, 2010), S122–129. ISSN: 1537-6591. DOI: 10.1086/651483. pmid: 20397940.

[31] European Study Group On Heterosexual Transmission Of HIV. “Comparison of Female to Male and Male to Female Transmission of HIV in 563 Stable Couples. European Study Group on Heterosexual Transmission of HIV.” In: *BMJ* 304.6830 (Mar. 28, 1992), pp. 809–813. ISSN: 0959-8138, 1468-5833. DOI: 10.1136/bmj.304.6830.809.

[32] Gary King et al. *Analyzing Incomplete Political Science Data: An Alternative Algorithm for Multiple Imputation*. Jan. 17, 2008. URL: https://papers.ssrn.com/abstract=1083698 (visited on 09/05/2023). preprint.
